# Fluorinated Hypoxia‐Responsive Aza‐BODIPY for NIR‐II FL/^19^F MR/PA Imaging and Phototherapy of Lung Cancer

**DOI:** 10.1002/advs.202521886

**Published:** 2026-01-18

**Authors:** Anfeng Li, Fang Wang, Mou Jiang, Yu Li, Xin Zhou, Zhong‐Xing Jiang

**Affiliations:** ^1^ State Key Laboratory of Magnetic Resonance Spectroscopy and Imaging National Center for Magnetic Resonance in Wuhan Wuhan Institute of Physics and Mathematics Innovation Academy for Precision Measurement Science and Technology Chinese Academy of Sciences‐Wuhan National Laboratory for Optoelectronics Huazhong University of Science and Technology Wuhan China; ^2^ University of Chinese Academy of Sciences Beijing China

**Keywords:** ^19^F MRI, NIR‐II fluorescence, phototherapy, redox‐responsive photosensitizer, tumor hypoxia

## Abstract

Tumor hypoxia limits the efficacy of photodynamic therapy (PDT), necessitating photosensitizers with hypoxia‐adaptive therapy and imaging. Here, we present a fluorinated *N*‐oxide aza‐BODIPY (**OFBD**) nanoemulsion (**OFBD‐NP**) for hypoxia‐responsive, multimodal imaging‐guided phototherapy. **OFBD** generates robust singlet oxygen under normoxia for PDT, but is reduced by CYP450 enzymes in hypoxic cells to photothermal‐potent **FBD**, enabling switchable PDT/PTT. Co‐assembly with fluorinated oil enhances oxygen delivery, boosts ^19^F MRI sensitivity, and promotes *J*‐aggregation, shifting fluorescence into the NIR‐II window for deep‐tissue imaging. The redox conversion also activates photoacoustic signals, enabling responsive tri‐modal imaging (NIR‐II FLI/^19^F MRI/PAI). **OFBD‐NP** shows potent cytotoxicity in vitro under both normoxic and hypoxic conditions via apoptosis. In vivo, it selectively accumulates in tumors, offers high‐contrast imaging, and leads to complete tumor regression in subcutaneous A549 models after laser irradiation, without systemic toxicity. This work demonstrates a smart nanoplatform that integrates deep‐tissue imaging and hypoxia‐triggered therapeutic switching, addressing major limitations of conventional photosensitizers in cancer imaging and phototherapy.

## Graphical abstract

Fluorinated *N*‐oxide aza‐BODIPY nanoemulsions integrate fluorination‐driven molecular design with co‐assembly in fluorinated oil for multimodal imaging‐guided phototherapy. The co‐assembly redshifts fluorescence into the NIR‐II window for deep‐tissue imaging and provides a unified ^19^F signal for background‐free, quantitative MRI. Under hypoxia, intracellular reduction switches photodynamic therapy to photothermal therapy and activates photoacoustic signals, achieving tri‐modal imaging‐guided adaptive phototherapy for efficient tumor regression.

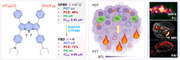

## 1 Introduction

Phototherapy, including photodynamic therapy (PDT) and photothermal therapy (PTT), has emerged as an effective and minimally invasive strategy for cancer treatment [1]. Its success relies on precise imaging guidance, efficient tumor accumulation, and localized activation of photosensitizers. However, current approaches are limited by poor imaging depth and tumor hypoxia [2, 3, 4]. Conventional fluorescence imaging (FLI) suffers from shallow tissue penetration and cannot effectively visualize deep‐seated tumors or guide therapy [5, 6, 7]. Meanwhile, the hypoxic tumor microenvironment (TME) impairs oxygen‐dependent type II PDT, leading to insufficient reactive oxygen species (ROS) generation and reduced efficacy [8, 9]. In addition, many PTT agents exhibit low photothermal conversion efficiency and poor photostability [10, 11]. Thus, robust photosensitizers with hypoxia‐adaptive therapeutic functions and multimodal imaging capabilities are urgently needed.

Several advanced modalities are capable of visualizing deep‐seated tumors. Incorporating these modalities into a photosensitizer enables multidimensional and accurate imaging guidance for therapy. Second near‐infrared window fluorescence imaging (NIR‐II FLI, 1000–1700 nm) offers high spatial resolution and low tissue scattering, allowing for deep‐tumor visualization [12, 13]. Fluorine‐19 magnetic resonance imaging (^19^F MRI) provides quantitative, background‐free imaging with high anatomical accuracy and excellent tissue penetration [14, 15, 16, 17]. Photoacoustic imaging (PAI) delivers real‐time visualization of light‐absorbing agents with greater depth than fluorescence‐based techniques [18, 19]. The combination of NIR‐II FLI, ^19^F MRI, and PAI yields a powerful multimodal imaging platform for noninvasive tracking and monitoring of tumors during treatment [20, 21]. On the therapeutic side, redox‐responsive photosensitizers that perform oxygen‐dependent type II PDT under normoxia and switch to oxygen‐independent type I PDT or PTT under hypoxia have emerged as promising tools to address tumor hypoxia [22, 23, 24]. However, such adaptive photosensitizers remain rare.

Aza‐BODIPY dyes are attractive for imaging and therapy due to their strong NIR absorption, photostability, and tunable properties [25, 26]. Yet, their tendency to aggregate and limited emission in the NIR‐II region restricts broader application. Recent work has aimed to address these limitations through structural modification [27, 28], *J*‐aggregation tuning [29, 30, 31, 32], and NIR‐II extension [33, 34, 35, 36]. In parallel, fluorination has emerged as an effective approach to impart ^19^F MRI capability and plasma membrane targeting [21, 37, 38], offering additional functionality beyond optical tuning. However, fluorinated aza‐BODIPY platforms that integrate hypoxia‐responsive PDT/PTT switching with multimodal imaging have not yet been explored.

**SCHEME 1:**
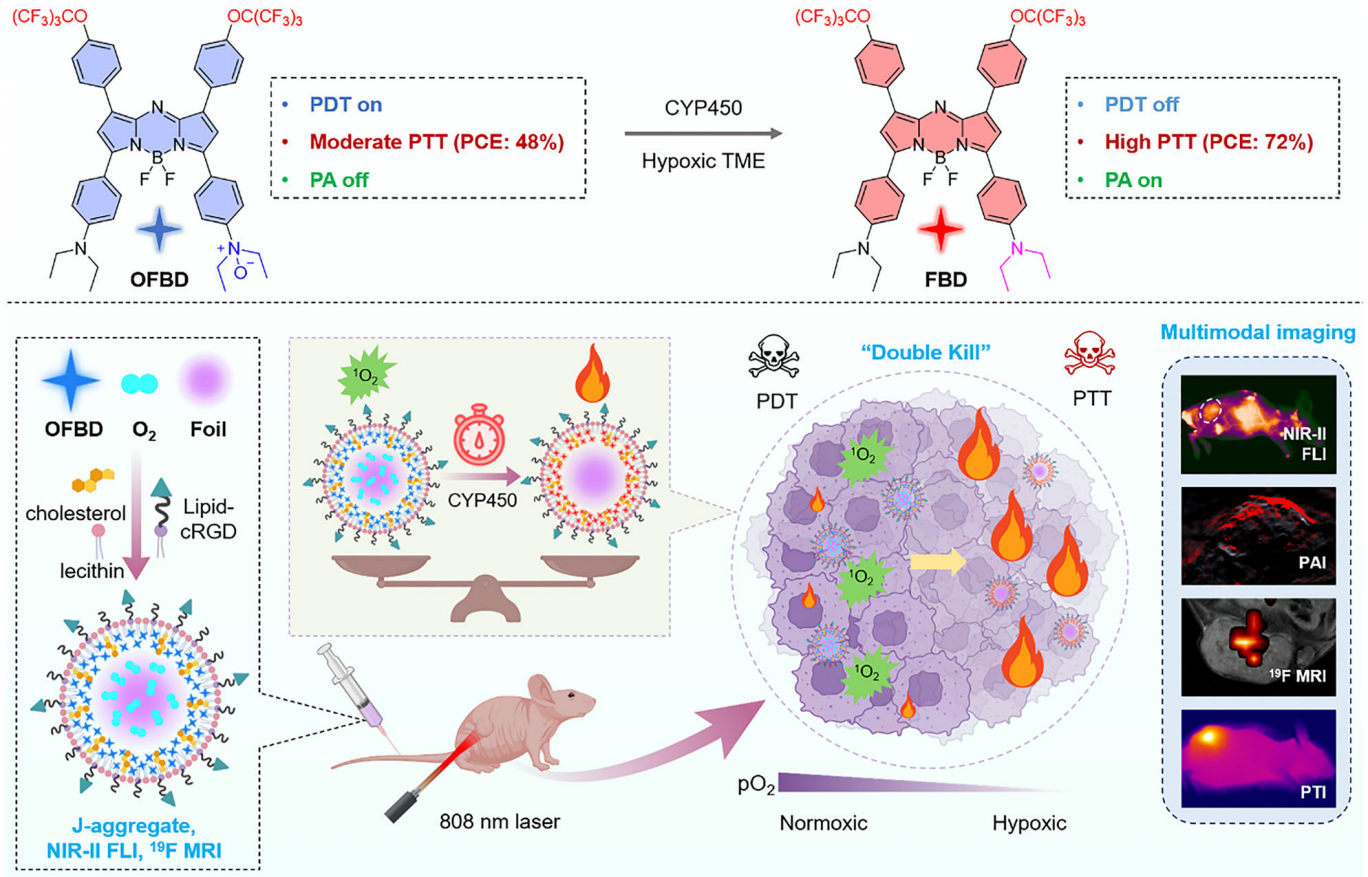
Fluorinated *N*‐oxide aza‐BODIPY (**OFBD**) for multimodal (NIR‐II FL/^19^F MR/PA) imaging‐guided, hypoxia‐responsive phototherapy of lung cancer.

Herein, we report a fluorinated *N*‐oxide aza‐BODIPY (**OFBD**) nanoemulsion (**OFBD‐NP**) that integrates NIR‐II FLI, ^19^F MRI, and PAI for multimodal imaging‐guided, hypoxia‐responsive phototherapy of lung cancer (Scheme 1). **OFBD** generates singlet oxygen (^1^O_2_) efficiently for type II PDT under normoxia, but is reduced by intracellular cytochrome P450 (CYP450) enzymes to fluorinated aza‐BODIPY (**FBD**) in hypoxic cells, switching to a potent photothermal agent and “turning on” PA signals. CYP450 are heme‐containing enzymes widely expressed in mammalian cells, including tumor cells. The hypoxia‐selective reduction is governed by the oxygen‐dependent catalytic mechanism of CYP450: under normoxia, molecular oxygen occupies the heme active site, limiting electron transfer to the *N*‐oxide, whereas hypoxia relieves this inhibition, allowing efficient conversion of **OFBD** to **FBD** [22, 39]. The co‐formulation of **OFBD** or **FBD** with fluorinated oil (**Foil**) facilitates the formation of *J*‐aggregates, extending fluorescence emission into the NIR‐II window. The shared perfluoro‐*tert*‐butyl (PFTB) group in **OFBD**, **FBD**, and **Foil** ensures that all fluorine atoms generate a single ^19^F NMR peak, leading to a well‐defined ^19^F MRI signal without splitting or chemical shift artifacts. This unified ^19^F signal, together with the oxygen‐carrying capability of **Foil** arising from its terminal PFTB groups that enhance oxygen solubility [40, 41], contributes to improved imaging sensitivity and PDT efficacy. Surface modification with cRGD enables tumor targeting via integrin α_v_β_3_ recognition, leading to enhanced accumulation of the nanoemulsion in tumors in vivo [42]. The resulting selective tumor accumulation, multimodal imaging guidance, and switchable “double‐kill” mechanism—via potent type II PDT followed by effective PTT—lead to complete tumor eradication. This multifunctional platform enables precise, real‐time monitoring of tumor progression and offers a powerful solution to overcome tumor hypoxia in cancer phototherapy.

## 2 Results and Discussion

### 2.1 Synthesis and Characterization of Photosensitizers

**FIGURE 1:**
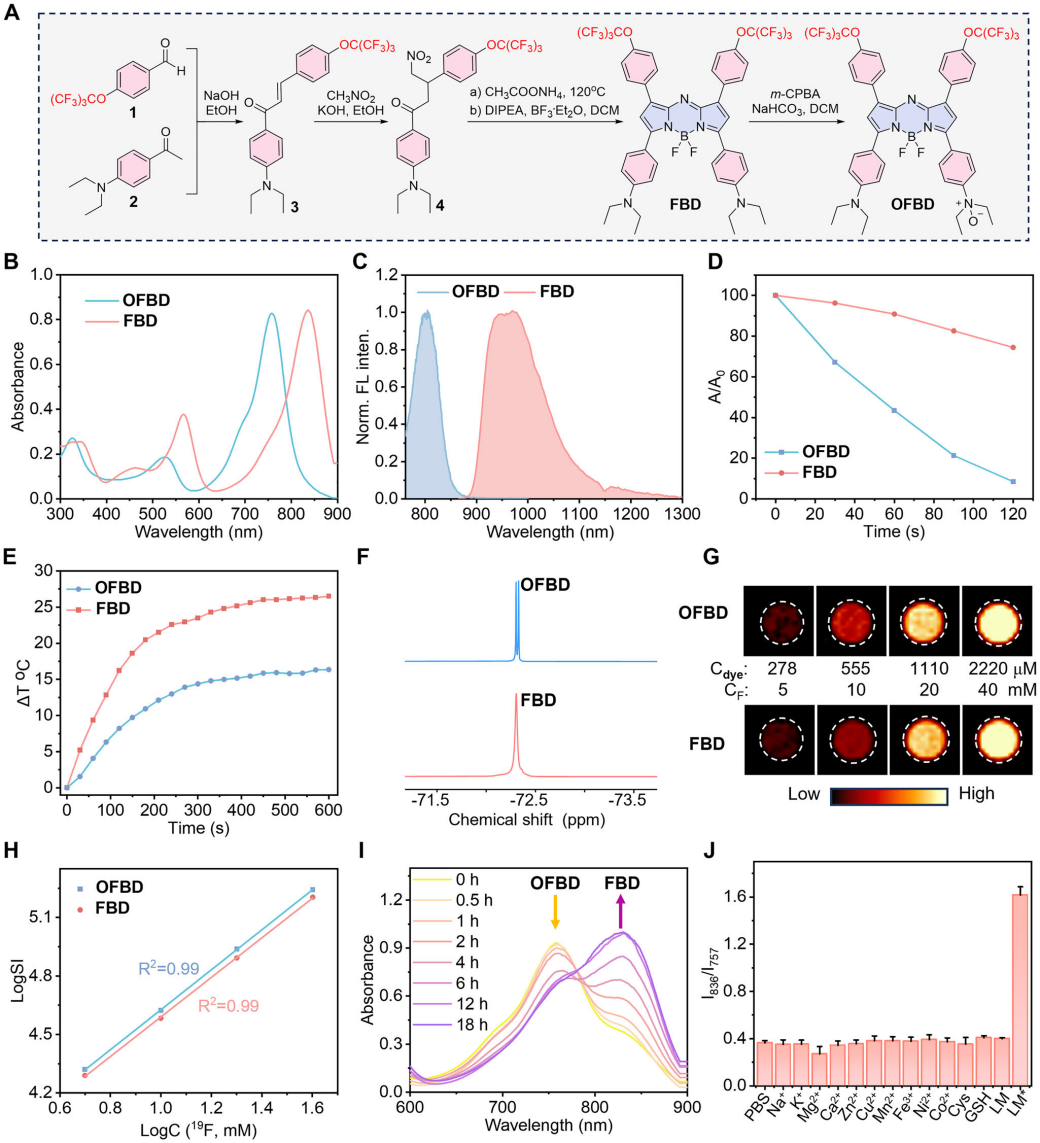
Synthetic scheme (A), absorption (B) and normalized emission (C) spectra of **OFBD** and **FBD**. Maximum absorption ratios of DPBF (60 µm) mixed with 2 µm **OFBD** or **FBD** (D). Temperature increases of 10 µm **OFBD** or **FBD** (E). Partial ^19^F NMR spectra (F), ^19^F MRI phantom images (G) and plot of LogSI vs. LogC(^19^F) (H) of **OFBD** and **FBD**. Incubation time‐dependent absorption spectra of **OFBD** in the presence of NADPH and CYP450 under hypoxic conditions (I). Maximum absorption ratios of **OFBD** incubated with metal ions, cysteine, GSH, and LM (J, ^*^under hypoxic conditions). Laser: 808 nm laser, 0.5 W cm^−2^. Solvent: chloroform for B–H, water for I–J.

**FBD** was synthesized following our previously reported protocol [37], yielding multi‐hundred milligram quantities with an overall 9% yield across three steps (Figure 1A). Selective oxidation using *m*‐CPBA [43] afforded **OFBD** in high yield. **FBD**, **OFBD**, and all intermediates were fully characterized by ^1^H/^13^C/^19^F NMR and mass spectrometry.

The optical properties of **FBD** and **OFBD** were evaluated in chloroform. Both showed marked redshifts in absorption compared to nonfluorinated analogs, with maxima at 836 nm (**FBD**) and 757 nm (**OFBD**). Their molar extinction coefficients (ε) were also enhanced, reaching 84050 and  83080 m ^−1^ cm^−1^, respectively (Figure 1B and Table S1), likely due to the electron‐donating effect of PFTB groups. Fluorescence spectra revealed further redshifts, with emission maxima at 971 nm for **FBD** and 807 nm for **OFBD**, extending well into the NIR region. Notably, **FBD** emission entered the NIR‐II window (Figure 1C), indicating strong potential for deep‐tissue imaging.

The photothermal and photodynamic properties of **FBD** and **OFBD** were systematically evaluated. Under 808 nm laser irradiation (0.5 W cm^−2^), 2 µm **OFBD** rapidly generated ^1^O_2_, as evidenced by complete depletion of the DPBF probe within 2 min. In contrast, **FBD** consumed only 28% of DPBF under identical conditions (Figure 1D; Figure S1), confirming the superior ROS generation of the oxidized derivative. Photothermal performance showed that 10 µm **FBD** induced a 26°C temperature rise after 10 min of irradiation, compared to 16°C for **OFBD** (Figure 1E), indicating higher photothermal conversion efficiency of **FBD**. These results demonstrate the complementary therapeutic profiles of the two compounds: **OFBD** is optimized for PDT, while **FBD** is more suitable for PTT. Photostability tests performed under a laser power density of 0.8 W cm^−2^ revealed no significant photobleaching of either **FBD** or **OFBD** after 210 s of laser irradiation, whereas ICG, a clinically used photosensitizer, showed a marked decrease in absorbance (Figure S2), highlighting the excellent photostability of the fluorinated aza‐BODIPY derivatives.

Density functional theory (DFT) calculations on **FBD** and **OFBD** were performed to assess the electronic influence of the *N*‐oxide moiety (Figure S3). The introduction of the *N*‐oxide moiety in **OFBD** significantly localizes the HOMO on the lower hemisphere of the molecule and increases the HOMO‐LUMO energy gap. This electronic reorganization provides a rationale for the experimentally observed blueshifted absorption and emission, as well as the enhanced PDT efficacy.

**FBD** gave a sharp ^19^F NMR singlet at −72.3 ppm in chloroform from its 18 magnetic equivalent fluorine atoms (Figure 1F). Upon oxidation, **OFBD** produced two distinct ^19^F peaks with close chemical shift reflecting symmetry loss, both suitable for ^19^F MRI. Both compounds were sensitively detectable by ^19^F MRI at 0.28 mm (equivalent to 5 mm fluorine) with a short acquisition time (256 s) and high SNR of 6.3 and 6.0, respectively (Figure 1G). A linear LogSI‐LogC(^19^F) relationship confirmed their potential for quantitative ^19^F MRI (Figure 1H).

To assess biotransformation, **OFBD** was incubated with liver microsomes (LM) and NADPH under hypoxic conditions for 18 h. Thin‐layer chromatography (TLC) analysis showed conversion of **OFBD** to **FBD** only under hypoxic conditions (Figure S4). This transformation was confirmed by UV–vis spectroscopy, which showed a decrease in **OFBD** absorption at 757 nm and a corresponding increase at 836 nm for **FBD** (Figure 1I). High‐performance liquid chromatography (HPLC) analysis further verified the hypoxia‐dependent reduction, with the well‐established CYP450 substrate AQ4N used as a positive control to support CYP450‐mediated activation (Figures S5 and S6) [44, 45]. In contrast, incubation with metal ions or biological reductants (e.g. cysteine, GSH) did not alter the 836/757 nm absorbance ratio (Figure 1J), indicating that CYP450 under hypoxia is required for the conversion. These results demonstrate that, despite the steric bulk of the PFTB groups, **OFBD** undergoes efficient enzymatic reduction to **FBD** under hypoxic, CYP450‐mediated conditions.

### 2.2 Formulation and Characterization of Nanoemulsions

**FIGURE 2:**
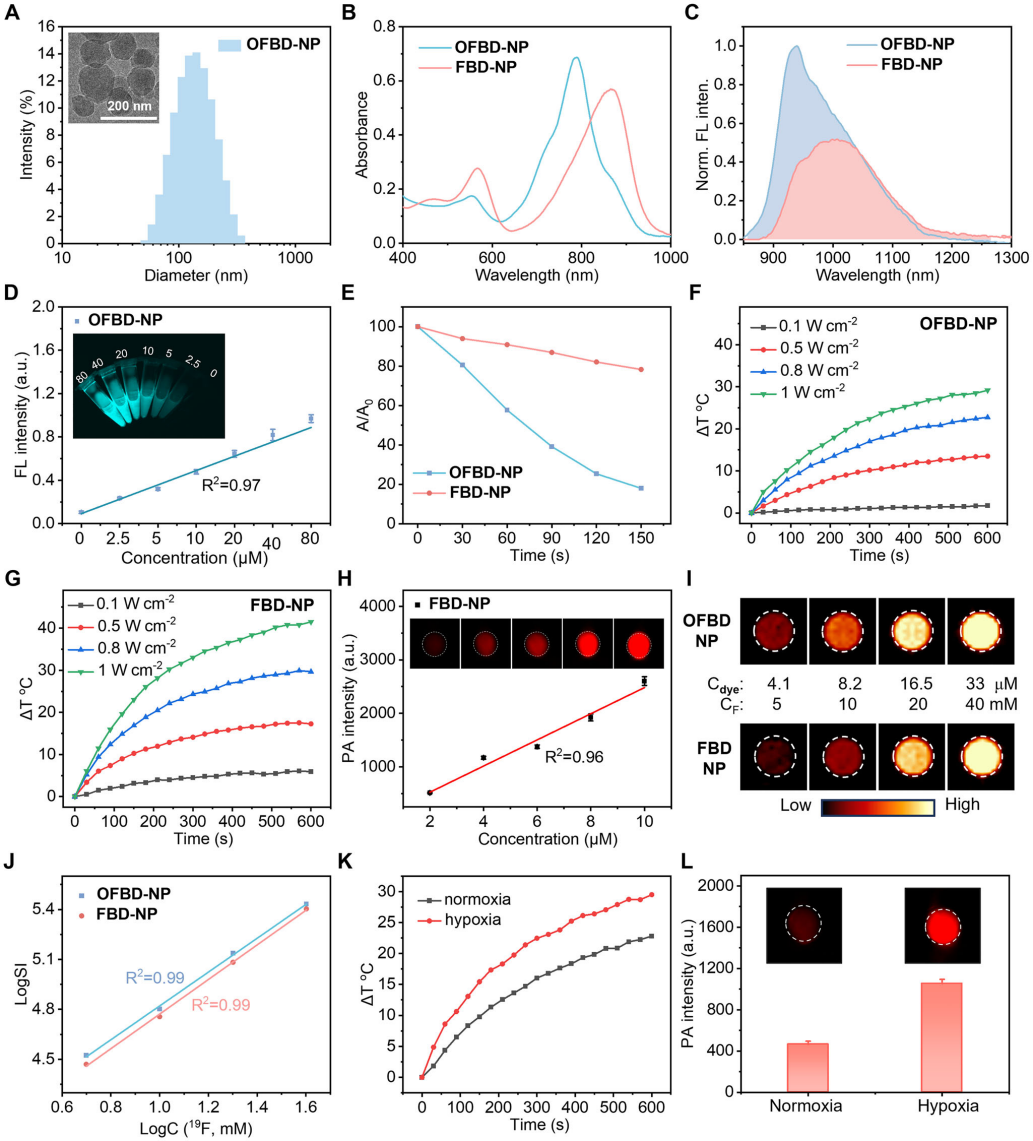
DLS with an inserted cryo‐TEM image of **OFBD‐NP** (A, scale bar: 200 nm). Absorption (B, 10 µm) and normalized emission (C, 10 µm) spectra of nanoemulsions. Plot of FL intensity vs. concentration with inserted images of **OFBD‐NP** (D). Absorption ratios of DPBF (60 µm) mixed with the nanoemulsions (E, 5 µm, 1 W cm^−2^) and temperature increases of **OFBD‐NP** (F) and **FBD‐NP** (G). Plot of PA signal intensity vs. **FBD‐NP** concentration with inserted PAI images (H). ^19^F MRI (I) and plot of LogSI vs. LogC(^19^F) (J) of the nanoemulsions. Temperature increases (K, 10 µm, 0.8 W cm^−2^) and PA signal intensity with inserted PAI images (L) of **OFBD‐NP** in the presence of CYP450 under hypoxic or normoxic conditions.

Due to their low water solubility, **OFBD** and **FBD** were formulated into nanoemulsions (**OFBD‐NP** and **FBD‐NP**) using **Foil**, lecithin (PL‐100 m), DSPE‐PEG_2000_, and cholesterol (Table S2). Dynamic light scattering (DLS) showed that **OFBD‐NP** and **FBD‐NP** had hydrodynamic diameters of 117 and 128 nm, respectively, with low PDIs (0.186 and 0.202; Figure 2A; Figure S7A). Cryo‐TEM confirmed spherical morphology. Zeta potential measurements revealed moderately negative surface charges of −24.6 mV for **OFBD‐NP** and −30.1 mV for **FBD‐NP**, suggesting good colloidal stability in aqueous media. Both nanoemulsions were stable in water, PBS, and DMEM with 10% FBS for at least 15 days (Figure S7).

Upon nanoemulsion formation, pronounced redshifts were observed in the optical spectra compared with the free photosensitizers. Specifically, the absorption maxima of **OFBD‐NP** and **FBD‐NP** were located at 790 and 872 nm, respectively, corresponding to redshifts of 33 nm for **OFBD** and 36 nm for **FBD** (Figure 2B; Figure S8). These spectral changes suggest the formation of *J*‐aggregates within the nanoemulsions [46, 47]. Consistently, the fluorescence emission maxima exhibited substantial redshifts, shifting from 807 to 939 nm for **OFBD** and from 971 to 1007 nm for **FBD** (Figure 2C), extending the emission further into the NIR‐II window. High‐resolution TEM (HRTEM) further revealed well‐defined lattice fringes within the nanoemulsions, with intermolecular spacings of approximately 3.0 Å for **OFBD‐NP** and 2.1 Å for **FBD‐NP** (Figure S8), indicating close *π*–*π* stacking between chromophores and supporting the formation of ordered aggregate structures [48]. Emission intensity correlated linearly with concentration (Figure 2D; Figure S9), and quantum yields were 0.63% (**OFBD‐NP**) and 0.51% (**FBD‐NP**, Figure S10).

Under 808 nm laser irradiation, 5 µm **OFBD‐NP** rapidly generated singlet oxygen, fully consuming DPBF in 150 s, whereas **FBD‐NP** showed limited ROS generation (Figure 2E; Figure S11). However, **FBD‐NP** exhibited superior photothermal activity, achieving a 40°C temperature rise in 10 min, with a photothermal conversion efficiency (PCE) of 72%, compared to 48% for **OFBD‐NP** (Figure 2F,G; Figure S12), indicating that reductive activation markedly enhances photothermal performance. Notably, the PCE of **FBD‐NP** also surpasses those of previously reported aza‐BODIPY derivatives, including BDP (40%) and BDP‐Oxide (12%) [23], as well as several representative hybrid or inorganic photothermal systems reported in the literature [49, 50]. Importantly, both nanoemulsions maintained stable absorption profiles and photothermal performance under five irradiation‐cooling cycles at a laser power density of 0.8 W cm^−2^ (Figures S2 and S12), indicating excellent photostability. These results confirm **OFBD‐NP**’s strong PDT efficacy and **FBD‐NP**’s dominant PTT capacity, demonstrating their complementary therapeutic potentials.

Photoacoustic (PA) analysis revealed that **FBD‐NP** produced a twofold higher signal than **OFBD‐NP** at equal concentrations (Figure 2H; Figure S13). PA signal intensity at 870 nm exhibited a linear correlation with **FBD‐NP** concentration (Figure 2H), supporting its use in quantitative PA imaging.

^19^F NMR showed that both nanoemulsions generated strong, unified fluorine signals due to the combined contributions of **Foil** and **OFBD/FBD** (Figure S14). They were detectable at 5 mm fluorine with an SNR of 9.6 and 8.8 in 256 s, respectively (Figure 2I). **Foil** further enhanced sensitivity, enabling detection of **OFBD** and **FBD** at much lower effective concentrations (4.1 µm). A linear LogSI‐LogC(^19^F) relationship confirmed their suitability for quantitative ^19^F MRI (Figure 2J). Despite the improved ^19^F MRI sensitivity enabled by **Foil** incorporation, the current detection limit remains below that required for clinical deep‐tissue imaging. Further sensitivity enhancement may be achieved through hyperpolarization, optimized coil design, or higher‐field MRI systems.

Biotransformation of **OFBD‐NP** was evaluated under hypoxic and CYP450 conditions. Upon irradiation, **OFBD‐NP** exhibited a significant temperature increase under hypoxia (Figure 2K), and its PA signal rose by 2.2‐fold compared to normoxia (Figure 2L), indicating conversion to **FBD‐NP**. This hypoxia‐responsive switch from PDT to PTT, along with enhanced PA output, confirms successful in situ transformation.

Therefore, nanoemulsion formulation of fluorinated aza‐BODIPYs induced *J*‐aggregate formation and extended NIR‐II fluorescence, enhanced ^19^F MRI sensitivity, and enabled hypoxia‐triggered phototherapeutic switching. **OFBD‐NP** thus functions as a tri‐modal agent for NIR‐II FLI/^19^F MRI/PAI‐guided hypoxia‐responsive cancer therapy.

### 2.3 In Vitro Multimodal Imaging and Phototherapy With OFBD‐NP

**FIGURE 3:**
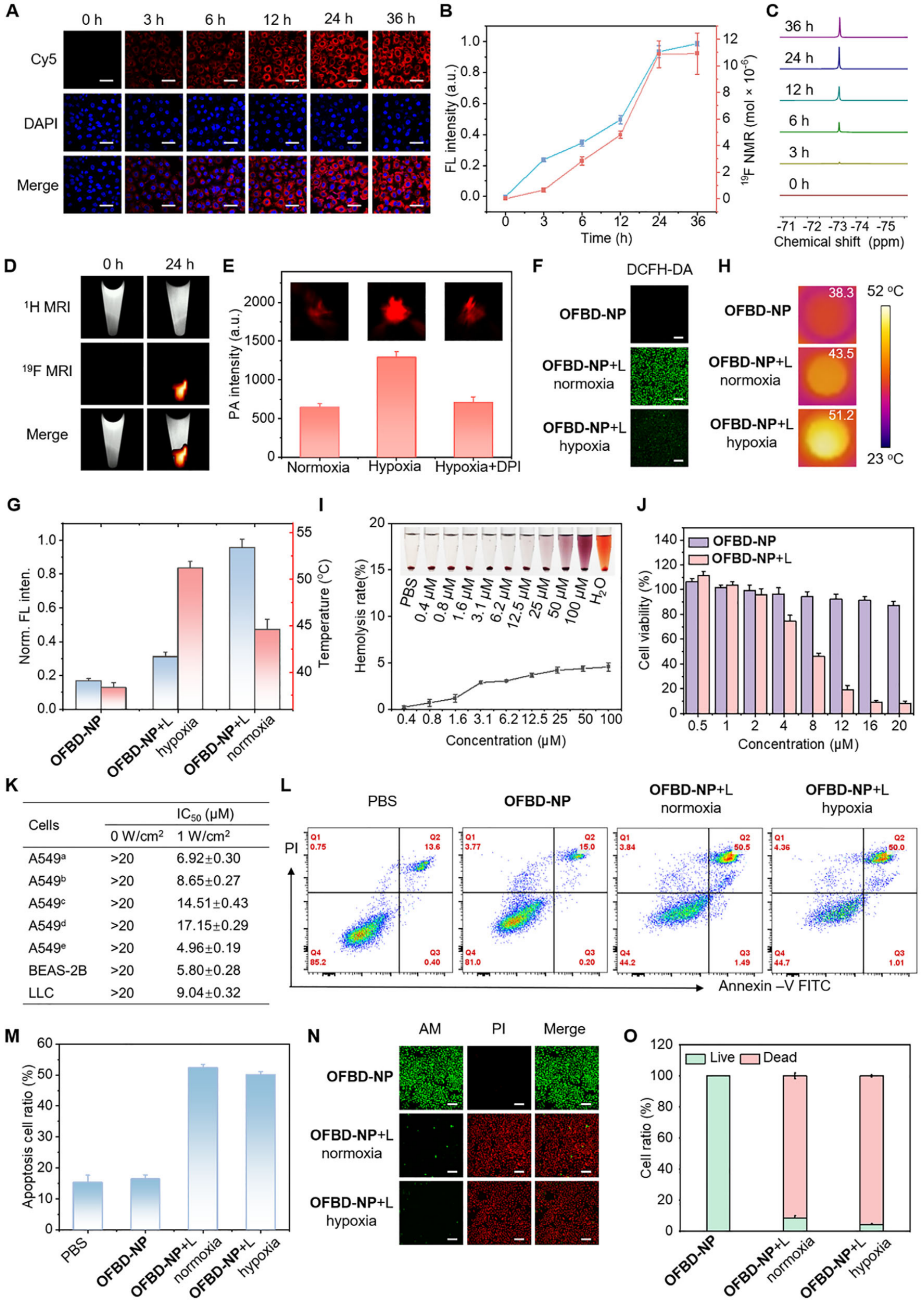
Time‐dependent CLSM images (A) and FL intensity (B) of **CyOFBD‐NP**‐treated cells. Time‐dependent ^19^F NMR (C) and ^19^F signal intensity (B), and ^19^F MRI (D) of **OFBD‐NP**‐treated cells. PA signal intensity and images of **OFBD‐NP**‐treated cells (E). DCFH‐DA‐stained CLSM images (F), photothermal images (H), and corresponding quantitative results (G) of **OFBD‐NP**‐treated cells. Hemolysis assay of **OFBD‐NP** with deionized water and PBS as controls (I). Cytotoxicity assay of **OFBD‐NP** (J). IC_50_ of the nanoemulsions (K, a: **OFBD‐NP**/normoxia, b: **OFBD‐NP**/hypoxia, c/d corresponded to a/b using soybean oil instead of **Foil**, e: **FBD‐NP**/normoxia). Flow cytometry (L) and apoptosis ratio (M) of **OFBD‐NP**‐treated cells. CLSM images (N) and dead cell ratio (O) of **OFBD‐NP**‐treated cells with AM/PI double staining. 808 nm laser at 1 W cm^−2^ was used in all cases. Scale bar is 50 µm for A and 100 µm for F, N. All concentrations refer to **OFBD**.

The cellular uptake of **OFBD‐NP** by human lung cancer A549 cells was evaluated using confocal laser scanning microscopy (CLSM). Due to the NIR fluorescence of **OFBD** being beyond the detection range of standard CLSM, **OFBD** was co‐loaded with DSPE‐PEG_2000_‐Cy5 to generate **CyOFBD‐NP** for imaging. Time‐dependent CLSM showed increasing Cy5 fluorescence in the cytoplasm, peaking at 24 h (Figure 3A,B). Quantitative ^19^F NMR analysis with sodium trifluoromethanesulfonate as an internal standard confirmed peak cellular uptake at 24 h (Figure 3B,C), consistent with “hot spot” ^19^F MRI results (Figure 3D). Approximately 1.9 × 10^12^ fluorine atoms were internalized per cell (Table S3), suitable for ^19^F MRI‐based cell tracking.

Bioreduction of **OFBD** in A549 cells was assessed via HPLC and PAI. Under normoxia (21% O_2_), only a portion of **OFBD** was converted to **FBD** (conversion: 68%), whereas under hypoxia (10% O_2_) the majority underwent reduction (conversion: 100%), indicating a positive correlation with hypoxia level (Figure S15). Consistently, PA signals increased approximately two‐fold under hypoxia, and were abolished by CYP450 inhibition with diphenyliodonium chloride (DPI), confirming CYP450‐mediated reduction of **OFBD** under hypoxia (Figure 3E).

Phototherapeutic efficacy was evaluated by measuring ROS generation and photothermal effects. Under normoxia, **OFBD‐NP**‐treated cells showed strong green fluorescence with DCFH‐DA (Figure 3F,G), indicating robust type II PDT activity. This signal was diminished under hypoxia. Photothermal imaging revealed low temperature elevation under normoxia, but a significant increase under hypoxia, indicating hypoxia‐triggered conversion of **OFBD** to **FBD** with enhanced photothermal performance (Figure 3H,G).

Biocompatibility studies showed <5% hemolysis at 100 µm **OFBD‐NP** (Figure 3I). CCK‐8 assays confirmed low dark cytotoxicity (> 20 µm) in A549, BEAS‐2B, and LLC cells, while laser‐irradiated cells showed high cytotoxicity (Figure 3J; Figure S16). IC_50_ values dropped significantly upon irradiation (Figure 3K), with **OFBD‐NP** showing potent phototoxicity under normoxic (IC_50_  =  6.92 µm) and hypoxic (IC_50_  =  8.65 µm) conditions, and **FBD‐NP** showing stronger photothermal cytotoxicity (IC_50_  =  4.96 µm). A control nanoemulsion using soybean oil (**SoyOFBD‐NP**) instead of **Foil** showed approximately twofold reduced phototoxicity, confirming **Foil**’s role in oxygen delivery and photosensitizer dispersion.

Flow cytometry revealed low cytotoxicity in the dark, but significant apoptosis under normoxia and hypoxia irradiation (Figure 3L,M), reflecting the dual mechanisms of PDT and PTT. Consistently, caspase‐3 activity assays showed markedly increased activity in the **OFBD‐NP** + laser group compared to the **OFBD‐NP** (without laser) and PBS control groups under both normoxic and hypoxic conditions (Figure S17), further confirming that apoptosis is a major mechanism of cell death induced by the treatment. Live/dead staining showed strong PI (red) and weak calcein‐AM (green) signals in laser and **OFBD‐NP**‐treated cells under both oxygen conditions (Figure 3N,O), indicating effective phototherapy.

Therefore, **OFBD‐NP** can be efficiently taken up by cancer cells, with uptake quantitatively monitored by ^19^F NMR and ^19^F MRI. Once internalized, hypoxia‐responsive bioreduction of **OFBD** to **FBD** activates PAI and switches the therapeutic mechanism from photodynamic to photothermal therapy, resulting in effective cancer cell elimination via apoptosis under both oxygen conditions and overcoming the limitations of traditional photosensitizers in hypoxic tumor environments.

### 2.4 In Vivo Biodistribution and Multimodal Imaging With OFBD‐NP

**FIGURE 4:**
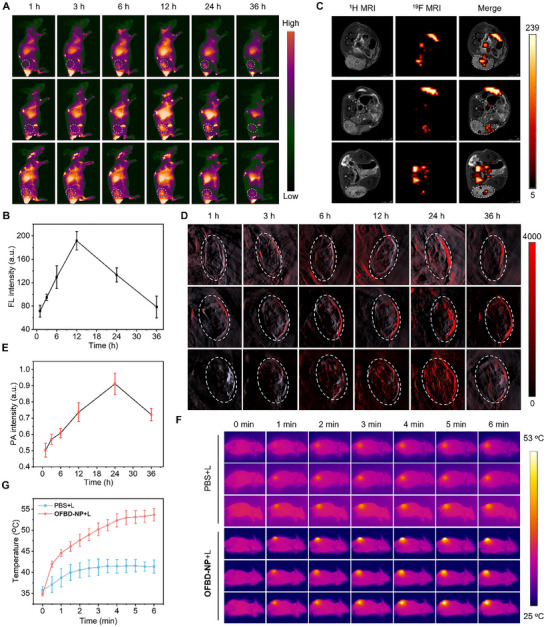
Time‐dependent NIR‐II FLI (A) and FL intensity in the tumor region (B), ^19^F MRI at 12 h (C), Time‐dependent PAI (870 nm) (D) and PA intensity in the tumor region (E), and photothermal imaging (F) and temperature (G) in the tumor region of mice with subcutaneous tumors after intravenous injection of **OFBD‐NP**.

Given the strong in vitro multimodal imaging performance, in vivo NIR‐II FLI, ^19^F MRI, PAI, and photothermal imaging (PTI) were conducted in BALB/c nude mice bearing subcutaneous A549 tumors. To enhance tumor targeting, the nanoemulsion was functionalized with DSPE‐PEG_2000_‐cRGD (2 mg/mL) to enable selective binding to α_v_β_3_ integrins overexpressed on A549 cells. The cRGD‐modified nanoemulsions retained their fundamental physicochemical and optical properties, as confirmed by DLS, UV–vis absorption, fluorescence spectroscopy, and concentration‐dependent fluorescence imaging. Cellular uptake studies using ^19^F NMR showed higher uptake for cRGD‐modified nanoemulsions, confirming enhanced tumor‐targeting capability (Figure S18). Following intravenous injection of **OFBD‐NP** (2 mg kg^−1^), NIR‐II FLI showed strong tumor signal, with enhanced accumulation compared to the non‐cRGD‐modified formulation, peaking at 12 h post‐injection (Figure 4A,B; Figure S19), indicating improved tumor targeting. At this time, “hot spot” ^19^F MRI confirmed fluorine signal localization in the tumor region (Figure 4C), demonstrating effective tumor accumulation of **OFBD‐NP**. PAI revealed a steady increase in PA signal intensity, reaching a maximum at 24 h (Figure 4D,E), which is consistent with the time‐dependent bioreduction of **OFBD** in the hypoxic tumor microenvironment following nanoemulsion accumulation. PTI showed a rapid temperature increase to 53 °C in the tumor site of **OFBD‐NP**‐treated mice after 6 min of laser irradiation, in contrast to the minimal heating observed in PBS‐treated controls (Figure 4F,G)—a temperature sufficient to induce tumor cell death. To further correlate this reduction with tumor hypoxia, we performed correlative analyses using tumors of different volumes. PAI at 870 nm was used to monitor the conversion of **OFBD** to **FBD**, with higher PA signal indicating more efficient reduction. Tumor tissues were sectioned and stained with the hypoxia‐specific probe pimonidazole, where higher red fluorescence intensity indicates greater hypoxia. In the smaller tumor (∼200 mm^3^), the 870 nm PA signal was lower, consistent with weaker pimonidazole fluorescence, indicating lower hypoxia and less **OFBD** reduction. In contrast, the larger tumor (∼400 mm^3^) showed stronger PA signal and more intense pimonidazole staining, reflecting higher hypoxia and increased **OFBD** bioreduction (Figure S20). Collectively, these results demonstrate that **OFBD‐NP** enables efficient tumor targeting and robust in vivo multimodal imaging, integrating NIR‐II FLI, ^19^F MRI, PAI, and PTI for image‐guided phototherapy. Based on its responsive imaging functionality, **OFBD‐NP** was therefore selected for subsequent in vivo theranostic evaluation.

### 2.5 In Vivo Phototherapy of Lung Cancer With OFBD‐NP

**FIGURE 5:**
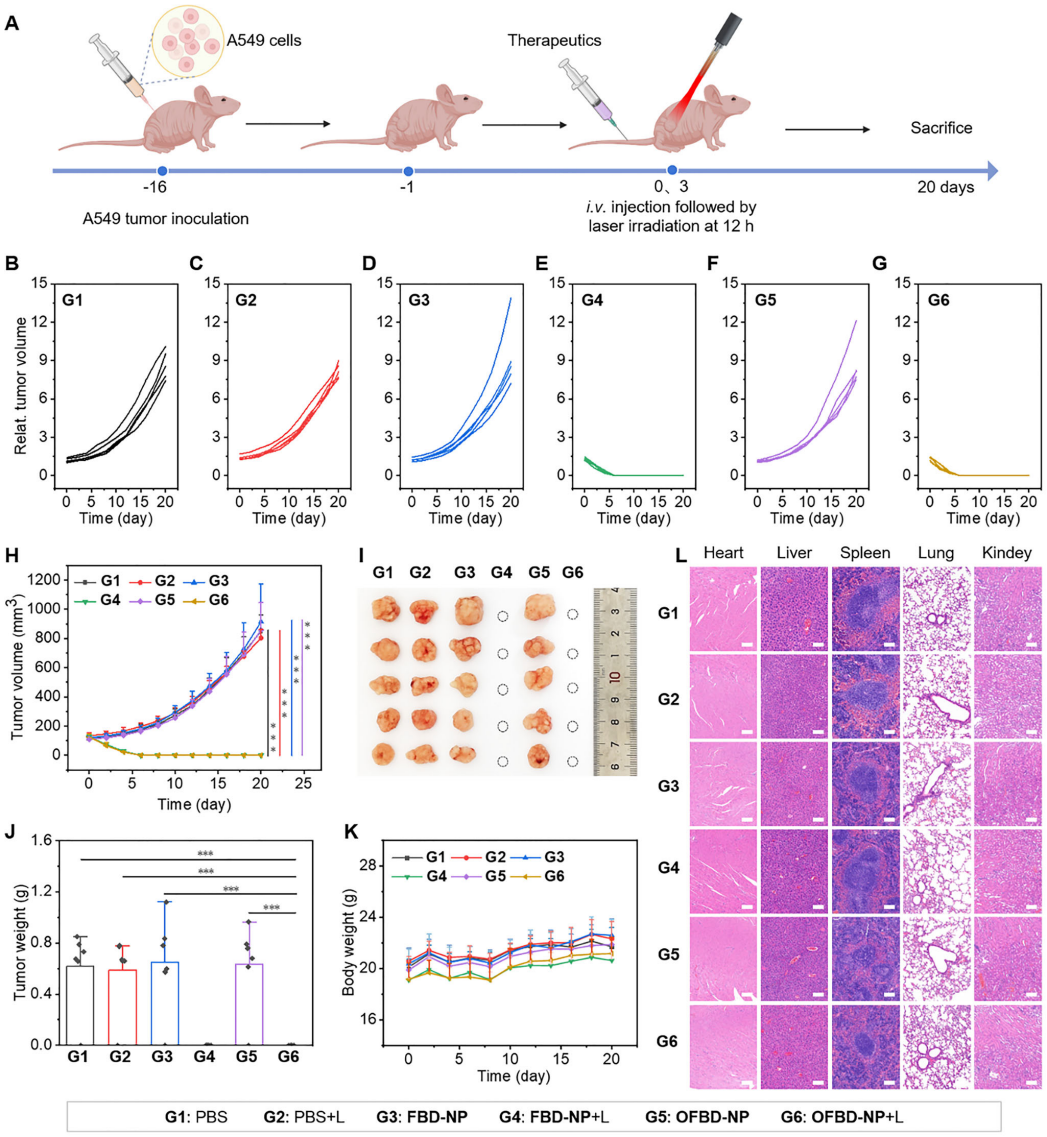
Schematic of phototherapy schedules (A). Grouped (B–G) and combined (H) tumor growth curves, tumor photographs (I), tumor weight (J), body weight curves (K), and H&E staining of harvested internal organs (L) of the treatment group. Scale bar is 100 µm. Data are presented as mean ± SD; *n* = 5, ^**^ *p* <0.01, ^***^ *p* <0.001.

The in vivo phototherapeutic efficacy of **OFBD‐NP** was assessed in a subcutaneous A549 lung cancer mouse model (Figure 5A). Once tumors reached ∼100 mm^3^, mice were randomly divided into six groups and treated with PBS, **FBD‐NP**, or **OFBD‐NP** (2 mg/kg), with or without laser irradiation (808 nm, 0.8 W cm^−2^, 6 min) administered 12 h post‐injection. Tumor growth was monitored every other day for 20 days. No significant tumor suppression was observed in the PBS, PBS+laser, **FBD‐NP**, or **OFBD‐NP** groups (Figure 5B–G). In contrast, both **FBD‐NP**+laser and **OFBD‐NP**+laser groups exhibited near‐complete tumor ablation, achieving a tumor growth inhibition (TGI) of 100% (Figure 5H). Macroscopic tumor evaluation and tumor weight measurements confirmed the absence of visible tumors in these groups, in stark contrast to the controls (Figure 5I,J). Notably, the **FBD‐NP**+laser group demonstrated complete tumor regression through photothermal therapy alone, highlighting the high efficacy of **FBD**‐mediated PTT. In comparison, the **OFBD‐NP**+laser group likely achieved tumor suppression through a synergistic “double‐kill” effect—initial PDT followed by PTT. This effect is supported by efficient tumor accumulation, as well as the robust and switchable photodynamic and photothermal activities of the formulations. While the PDT contribution cannot be directly distinguished in this model, it may complement PTT and enhance therapeutic outcomes. Future studies will further investigate the advantages of PDT‐PTT combination. Additionally, enhanced oxygen delivery from the fluorinated photosensitizer and **Foil** nanodroplet helped alleviate tumor hypoxia, improving PDT outcomes.

No significant changes in body weight were observed throughout the study, indicating good systemic tolerance (Figure 5K). Histological analysis of major organs revealed no detectable tissue damage, confirming the biocompatibility of the treatments (Figure 5L). Overall, these results demonstrate that both **OFBD‐NP** and **FBD‐NP** effectively enhance oxygenation, and enable potent, image‐guided phototherapy—leading to pronounced tumor regression, suppressed proliferation, and increased apoptotic cell death.

## 3 Conclusions

In summary, we have developed a fluorinated aza‐BODIPY nanoemulsion (**OFBD‐NP**) capable of overcoming tumor hypoxia through a redox‐responsive switch between PDT and PTT. This transformation is triggered by intracellular CYP450 under hypoxic conditions, enabling a synergistic “double‐kill” phototherapeutic effect. **OFBD‐NP** exhibits multimodal imaging capabilities, including NIR‐II fluorescence, ^19^F MR, PA, and photothermal imaging, facilitating accurate tumor localization and image‐guided therapy. The use of a fluorinated oil core (**Foil**) further improves ^19^F MRI sensitivity and oxygen delivery, enhancing PDT efficacy. Both in vitro and in vivo results demonstrate potent and safe tumor regression. Compared with previously reported hypoxia‐responsive photosensitizer systems, this work provides a rational design for smart nanotheranostics that integrates deep‐tissue, background‐free multimodal imaging with hypoxia‐adaptive phototherapy within a single nanosystem, offering a strategy to overcome limitations in imaging depth. A limitation of this study is the use of subcutaneous tumors, which may not fully reflect clinical tumor complexity. Future work should explore orthotopic models and enhance ^19^F MRI sensitivity to enable real‐time therapeutic monitoring and broader clinical translation.

## 4 Experimental Section/Methods

### 4.1 Materials and General Information

Egg yolk lecithin (PL‐100 m) was purchased from Aiweituo Medicine Technology Co., Ltd. Cholesterol and DSPE‐PEG_2000_ were purchased from Adamas. Medicinal‐grade soybean oil was purchased from Aladdin. SOSG was purchased from MeilunBio. DCFH‐DA was purchased from Meryer. CCK‐8, DAPI, Calcein/PI cell viability/cytotoxicity assay kit, 4% paraformaldehyde fix solution (4% PFA) were all purchased from Beyotime. Rat liver microsomes, NADPH and diphenyliodonium chloride (DPI) were purchased from Sigma–Aldrich. AQ4N was purchased from MedChemExpress. Hypoxyprobe Plus Kit was purchased from Hypoxyprobe.

^1^H, ^13^C, and ^19^F NMR spectra were recorded on a Bruker 400, 500, or 600 MHz spectrometer. ^1^H NMR spectra were referenced to tetramethylsilane (s, 0.00 ppm) using CDCl_3_ as solvent. ^13^C NMR spectra were referenced to solvent carbons (77.16 ppm). ^19^F NMR spectra were referenced to 2% hexafluorobenzene (s, −164.90 ppm) in CDCl_3_. The splitting patterns for ^1^H NMR and ^19^F NMR spectra were denoted as follows: s = singlet, d = doublet, t = triplet, q = quartet, m = multiplet. High‐resolution mass spectra (HRMS) were recorded on a Thermo Fisher Scientific Q Exactive Focus. Flash chromatography was performed on 200–300 mesh silica gel with ethyl acetate/petroleum ether as eluent. The UV–vis absorption and fluorescence emission spectra were measured using a UV‐2600 UV–vis spectrophotometer (Shimadzu, Japan) and an F‐4700 spectrofluorophotometer (Hitachi, Japan), respectively. The size distribution and polymer dispersion index (PDI) of nanoemulsion were determined by a dynamic light scattering (DLS) instrument (Malvern, UK). An 808 nm laser was used for photothermal conversion and ROS generation experiments. Confocal laser scanning microscopy imaging was performed on a Nikon confocal laser scanning microscope (CLSM). The apoptosis assay was performed using a flow cytometer (Beckman CytoFLEX‐S, USA). ^19^F MRI was performed on a Bruker BioSpec 9.4T MRI system. Small animal fluorescence imaging was carried out by the NIR‐II fluorescence imaging system (NIROPTICS, China). Photothermal imaging was measured with a thermal imaging camera (Zhejiang Dali Technology, China). The PAI experiment was carried out on a real‐time multispectral photoacoustic tomography system (iThera Medical, Germany).

### 4.2 Preparation of Nanoemulsions

Preparation of **OFBD‐NP**. Egg yolk lecithin (15 mg), cholesterol (3 mg), DSPE‐PEG_2000_ (1 mg), **Foil** (30 mg), and **OFBD** (0.6 mg) were dissolved in DCM and evaporated under reduced pressure. Subsequently, 1 mL of deionized water was added, followed by sonication for 15 min. The mixture was then extruded 15 times through a 200 nm polycarbonate membrane filter to obtain **OFBD‐NP**. **FBD‐NP** were prepared using the same procedure.

### 4.3 Biotransformation of **OFBD** Under Normoxic and Hypoxic Conditions

Phosphate buffer (pH *7.4*) was degassed by bubbling with nitrogen for 30 min before use. **OFBD** was dissolved in deionized water containing 0.1% (v/v) cremophor EL (CrEL) and incubated with liver microsomes (LM, 200 µg/mL) and NADPH (100 µg/mL) at 37°C. Incubations were carried out under either normoxic (21% O_2_) or hypoxic (N_2_ atmosphere) conditions and terminated at predetermined time points (0, 0.5, 1, 2, 4, 6, 12, and 18 h). After incubation, the reaction mixture was extracted with DCM, and the organic phase was collected and concentrated under reduced pressure. The residue was redissolved in chloroform, and UV–vis absorption spectra were recorded to evaluate hypoxia‐dependent spectral changes associated with the biotransformation of **OFBD**.

### 4.4 Cellular Bioreduction in A549 Cells

A549 cells were seeded in 10 cm culture dishes and allowed to adhere under normoxic (21% O_2_) or hypoxic (10% O_2_) conditions. The cells were then incubated with **OFBD‐NP** (+ cRGD) for 12 h under the corresponding oxygen conditions. After incubation, cells were collected, washed three times with PBS, and lysed using RIPA buffer for 15 min. The lysates were treated with tetrahydrofuran to facilitate the dissolution of **OFBD** and its reduced products, followed by HPLC analysis to quantify the conversion of **OFBD** to **FBD**. In parallel, cell pellets collected after incubation were used directly for photoacoustic imaging to assess hypoxia‐dependent signal changes.,

### 4.5 In Vivo NIR‐II Fluorescence Imaging

Mice bearing A549 tumors were intravenously injected with 100 µL of **OFBD‐NP** (C**_OFBD_** = 2 mg/kg). Biodistribution of **OFBD‐NP** was monitored using a NIR‐II fluorescence imaging system (Series II 900/1700, NIROPTICS, China) at 1, 3, 6, 12, 24, and 36 h post‐injection.

### 4.6 In Vivo ^19^F MRI

Mice bearing A549 tumors received intravenous injections of 200 µL of **OFBD‐NP** (C**_OFBD_** = 4 mg/kg, C_F_ = 9 mmol/kg). ^19^F MRI was performed using the RARE sequence with the following parameters: center frequency = 376.527883 MHz, TR = 1500 ms, TE = 3 ms, FOV = 37 mm × 37 mm, SI = 15 mm, matrix size = 32 × 32, rare factor = 8, and number of averages = 600. Anatomical reference ^1^H MR images were acquired using the RARE sequence with the following parameters: TR = 4000 ms, TE = 11 ms, FOV = 30 mm × 30 mm, SI = 1 mm, matrix size = 256 × 256, rare factor = 8, and number of averages = 4.

### 4.7 In Vivo PAI

Mice bearing A549 tumors were intravenously injected with 100 µL of **OFBD‐NP** (C**_OFBD_** = 2 mg/kg). Tumor PA intensity was measured at 1, 3, 6, 12, 24, and 36 h post‐injection.

### 4.8 Bioreduction of **OFBD** in Vivo

Tumor‐bearing mice with tumor volumes of approximately 200 and 400 mm^3^ were selected and intravenously injected with **OFBD‐NP** (+cRGD) via the tail vein. Photoacoustic imaging (PAI) was performed 24 h post‐injection to monitor hypoxia‐dependent bioreduction of **OFBD** in tumors of different sizes.

### 4.9 Assessment of Tumor Hypoxia in Tumor Tissues

Tumor hypoxia levels were evaluated using the Hypoxyprobe Plus Kit. Following photoacoustic imaging, mice were intravenously injected with pimonidazole hydrochloride (100 µL, 12 mg mL^−1^). The mice were then sacrificed, and tumor tissues were harvested, embedded, and cryosectioned into 20 µm slices. Tumor sections were stained with a FITC‐labeled mouse IgG1 monoclonal antibody (FITC‐Mab1) according to the manufacturer's protocol and imaged by confocal laser scanning microscopy (CLSM). Fluorescence signals from pimonidazole adducts were collected in the 500–550 nm range under 488 nm excitation.

### 4.10 Statistical Analysis

Data are presented as mean ± standard deviation from *n* ≥3 replicates. Statistical significance was evaluated using an unpaired two‐sided Student's *t*‐test, with p values calculated in Microsoft Excel. Asterisks indicate significant differences (^*^ *p* <0.05, ^**^ *p* <0.01, ^***^ *p* <0.001).

## Conflicts of Interest

The authors declare no conflicts of interest.

## Supporting information




**Supporting File**: advs73857‐sup‐0001‐SuppMat.docx.

## Data Availability

The data that support the findings of this study are available in the supplementary material of this article.
