# Supplementary material for: Fluorinated Hypoxia‐Responsive Aza‐BODIPY for NIR‐II FL/19F MR/PA Imaging and Phototherapy of Lung Cancer
Source: Adv Sci (Weinh). 2026 Jan 18;13(17):e21886. doi: 10.1002/advs.202521886 (PMC13042524; doi:10.1002/advs.202521886)
Supplement: Supplementary file 1 — Supporting File: advs73857‐sup‐0001‐SuppMat.docx. [file ADVS-13-e21886-s001.docx]

Supporting Information

**Fluorinated Hypoxia-Responsive Aza-BODIPY for NIR-II FL/^19^F MR/PA Imaging and Phototherapy of Lung Cancer**

Anfeng Li,^a,#^ Fang Wang,^a,#^ Mou Jiang,^a,#^ Yu Li,^a,b*^ Xin Zhou,^a,b*^ and Zhong-Xing Jiang^a,b*^

^a^ State Key Laboratory of Magnetic Resonance Spectroscopy and Imaging, National Center for Magnetic Resonance in Wuhan, Wuhan Institute of Physics and Mathematics, Innovation Academy for Precision Measurement Science and Technology, Chinese Academy of Sciences-Wuhan National Laboratory for Optoelectronics, Huazhong University of Science and Technology, Wuhan 430071, China.

^b^ University of Chinese Academy of Sciences, Beijing 100049, China.

^*^ Email: zxjiang@apm.ac.cn (Z.-X. Jiang), xinzhou@wipm.ac.cn (X. Zhou), liyu@apm.ac.cn (Y. Li)

^#^ A. Li., F. Wang., and M. Jiang contributed equally to this work

**Table of Contents**

[1 Supplementary figures and tables 3](#_Toc6060)

[2 Synthesis and characterization of **FBD** and **OFBD** 18](#_Toc2724)

[2.1 Synthetic procedures 18](#_Toc21368)

[2.2 Photothermal performance and photostability of **OFBD** and **FBD** 21](#_Toc6325)

[2.3 Detection of singlet oxygen generation via DPBF degradation 21](#_Toc6912)

[2.4 Biotransformation of **OFBD** under normoxic and hypoxic conditions 22](#_Toc19085)

[2.5 Preparation of nanoemulsions 22](#_Toc21320)

[2.6 Determination of photothermal conversion efficiency 23](#_Toc735)

[2.7 *In vitro* ^19^F MRI 23](#_Toc9974)

[3 Cell culture 24](#_Toc12683)

[3.1 Intracellular uptake 24](#_Toc16114)

[3.2 Cellular bioreduction in A549 cells 24](#_Toc6713)

[3.3 *In vitro* cytotoxicity assays 25](#_Toc7451)

[3.4 Live/dead cell staining assays 25](#_Toc14531)

[3.5 Intracellular ROS imaging in A549 cells 25](#_Toc4495)

[3.6 Hemolysis assay 26](#_Toc31182)

[3.7 Apoptosis assay 26](#_Toc18269)

[4 *In vivo* assays 26](#_Toc28991)

[4.1 Bioreduction of **OFBD** in vivo 27](#_Toc2967)

[4.2 Assessment of tumor hypoxia in tumor tissues 27](#_Toc17794)

[4.3 *In vivo* NIR-II fluorescence imaging 27](#_Toc18973)

[4.4 *In vivo* ^19^F MRI 27](#_Toc32170)

[4.5 *In vivo* PAI 28](#_Toc21913)

[4.6 Therapeutic efficacy evaluation 28](#_Toc4600)

[5 Statistical analysis 28](#_Toc31877)

[6 ^1^H/^19^F/^13^C NMR and HRMS spectra of compounds 29](#_Toc22868)

[7 References 43](#_Toc15152)

# 1 Supplementary figures and tables


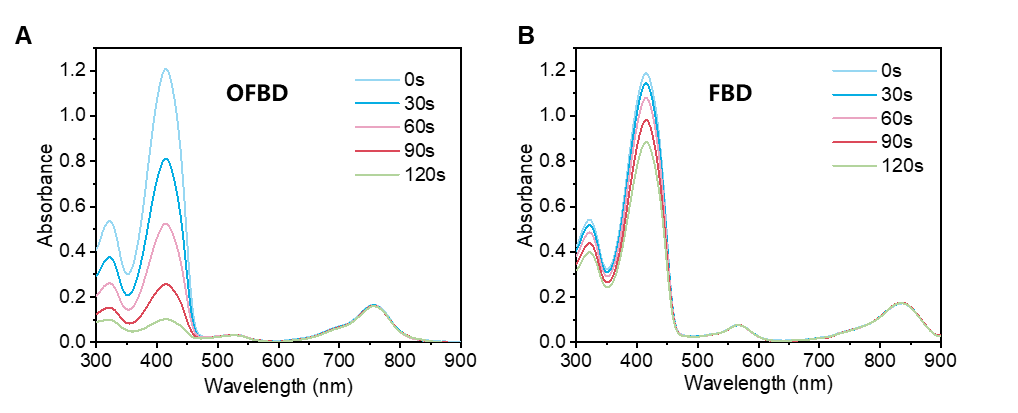


**Figure S1**. Time-dependent absorption spectra of DPBF (60 µM) mixed with either **OFBD** (A, 2 µM) or **FBD** (B, 2 µM) in CHCl_3_ under 808 nm laser irradiation (0.5 W cm^-2^).


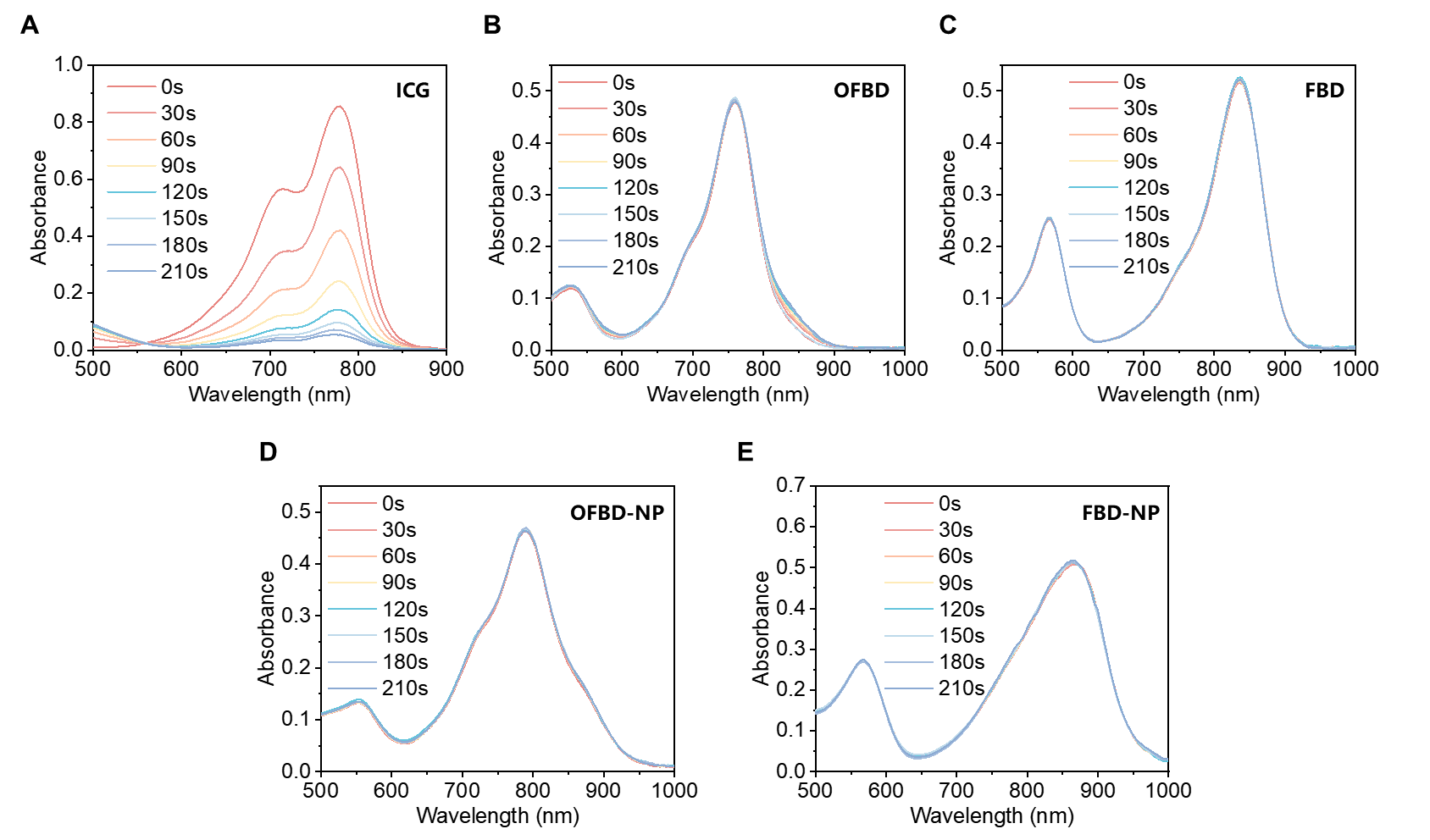


**Figure S2**. Time-dependent absorption spectra of **ICG** (A), **OFBD** (B), **FBD** (C), **OFBD-NP** (D), and **FBD-NP** (E) under 808 nm laser irradiation (0.8 W cm^-2^).


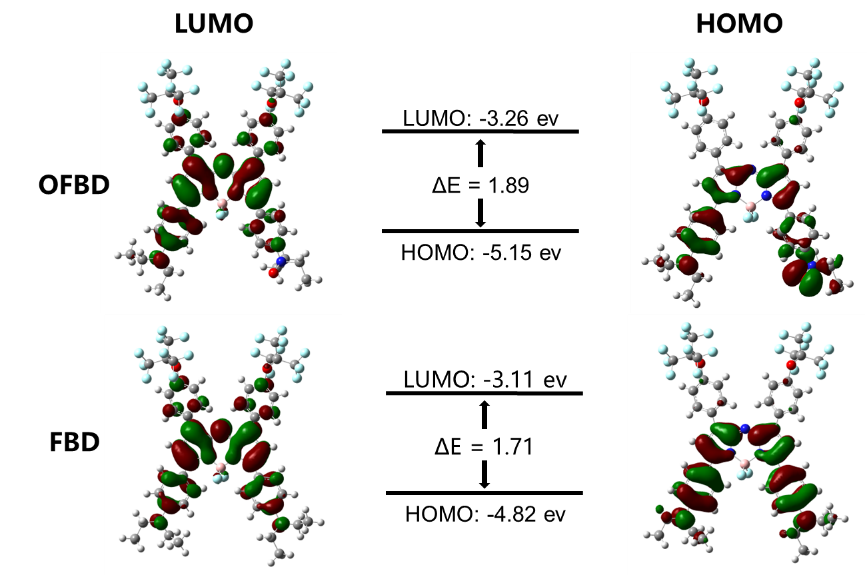


**Figure S3**. Calculated HOMO and LUMO energy levels of **OFBD** and **FBD** using Gaussian 16W at the B3LYP/6-31G(d) level.


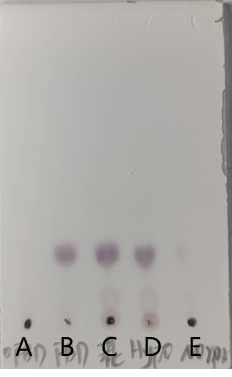


**Figure S4**. Thin layer chromatography (TLC) analysis of **OFBD** treated with NADPH and CYP450 for 18 h under hypoxia. Spots: (A) **OFBD**; (B) **FBD**; (C) co-spot of **OFBD** and **FBD**; (D) reaction mixture under hypoxia; (E) reaction mixture under normoxia. Eluent: petroleum ether/ethyl acetate (10:1, v/v).


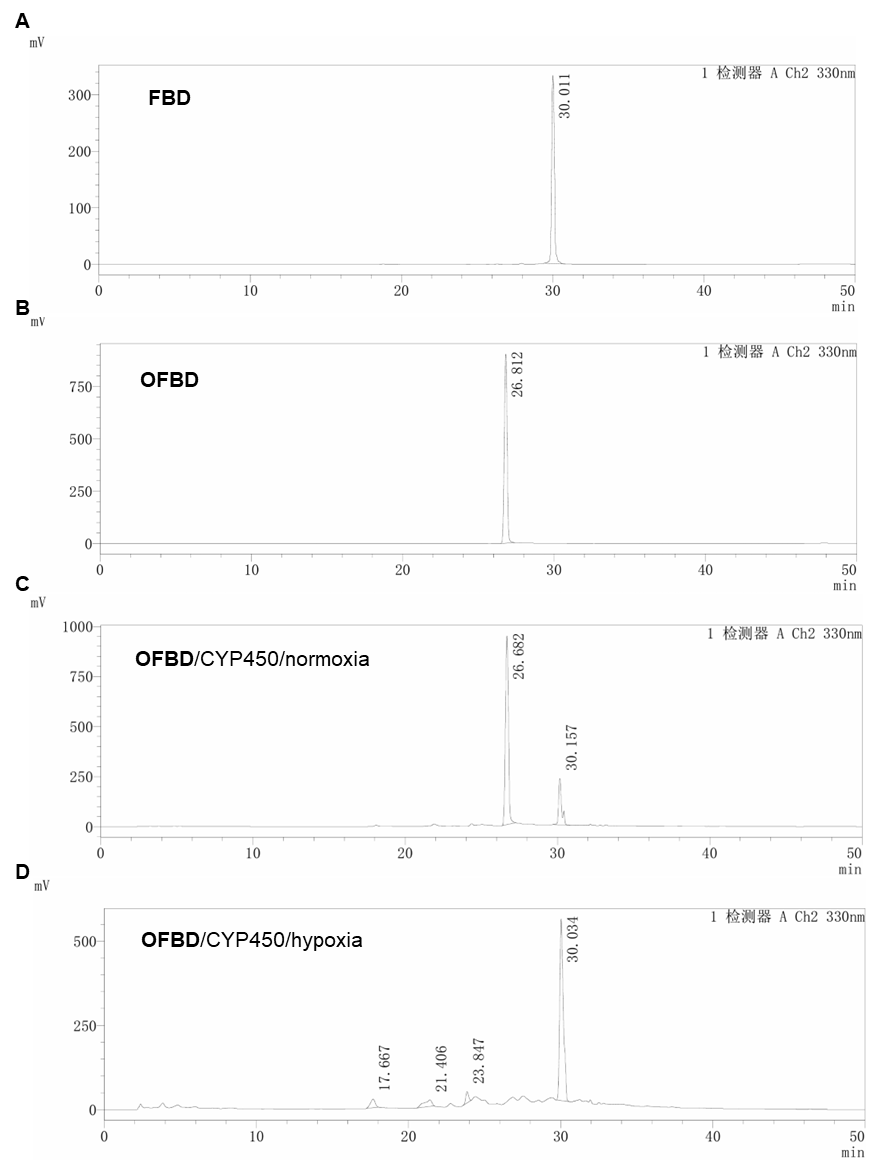


**Figure S5**. High performance liquid chromatograms of **FBD** (A), **OFBD** (B), **OFBD** treated with NADPH and CYP450 for 18 h under normoxic (C) or hypoxic (D) conditions.


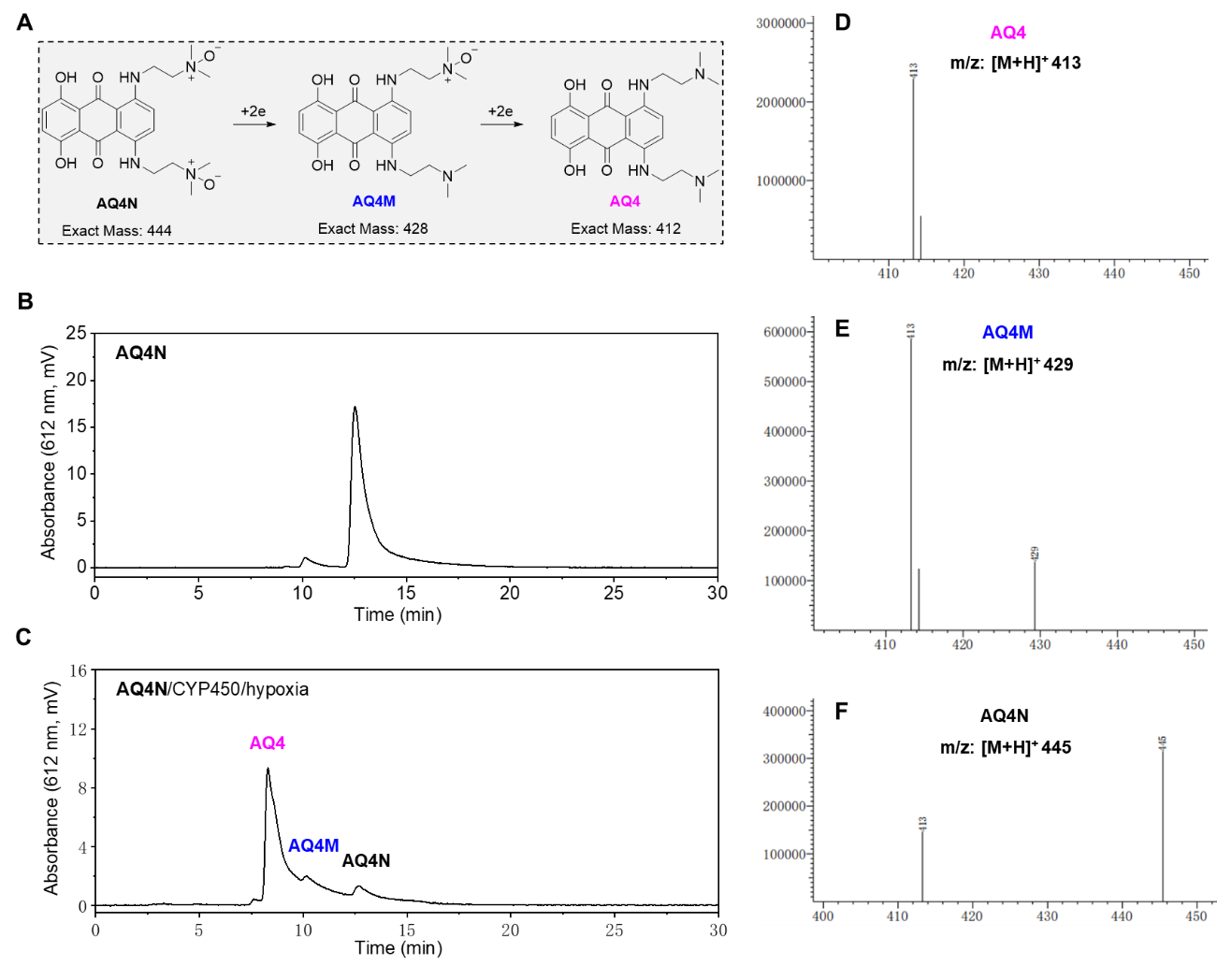


**Figure S6**. Schematic illustration of the reduction of **AQ4N** to **AQ4** via the singly reduced intermediate **AQ4M** (A). HPLC profile of **AQ4N** (B). HPLC profile of **AQ4N** after incubation with NADPH and CYP450 under hypoxic condition for 2 h (C). Mass spectra of **AQ4**, **AQ4M**, and residual **AQ4N** (D-F), respectively, corresponding to peaks in panel (C).


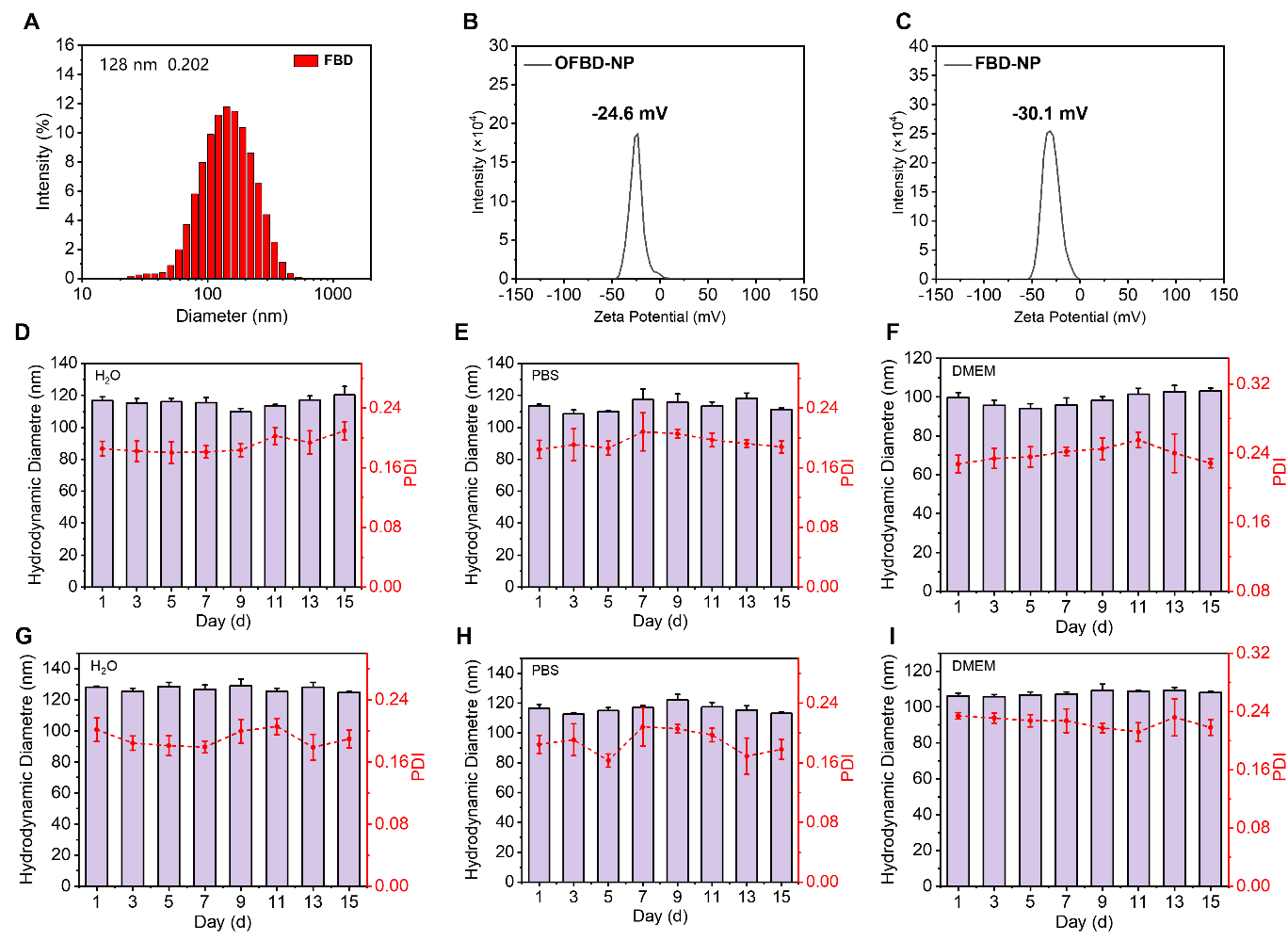


**Figure S7**. DLS of **FBD-NP** (A). Zeta potentials of **OFBD-NP** (B) and **FBD-NP** (C). The hydrodynamic diameters and polydispersity index (PDI) values of **OFBD-NP** (D-F) and **FBD-NP** (G-I) in H_2_O, PBS, and DMEM over 15 days.


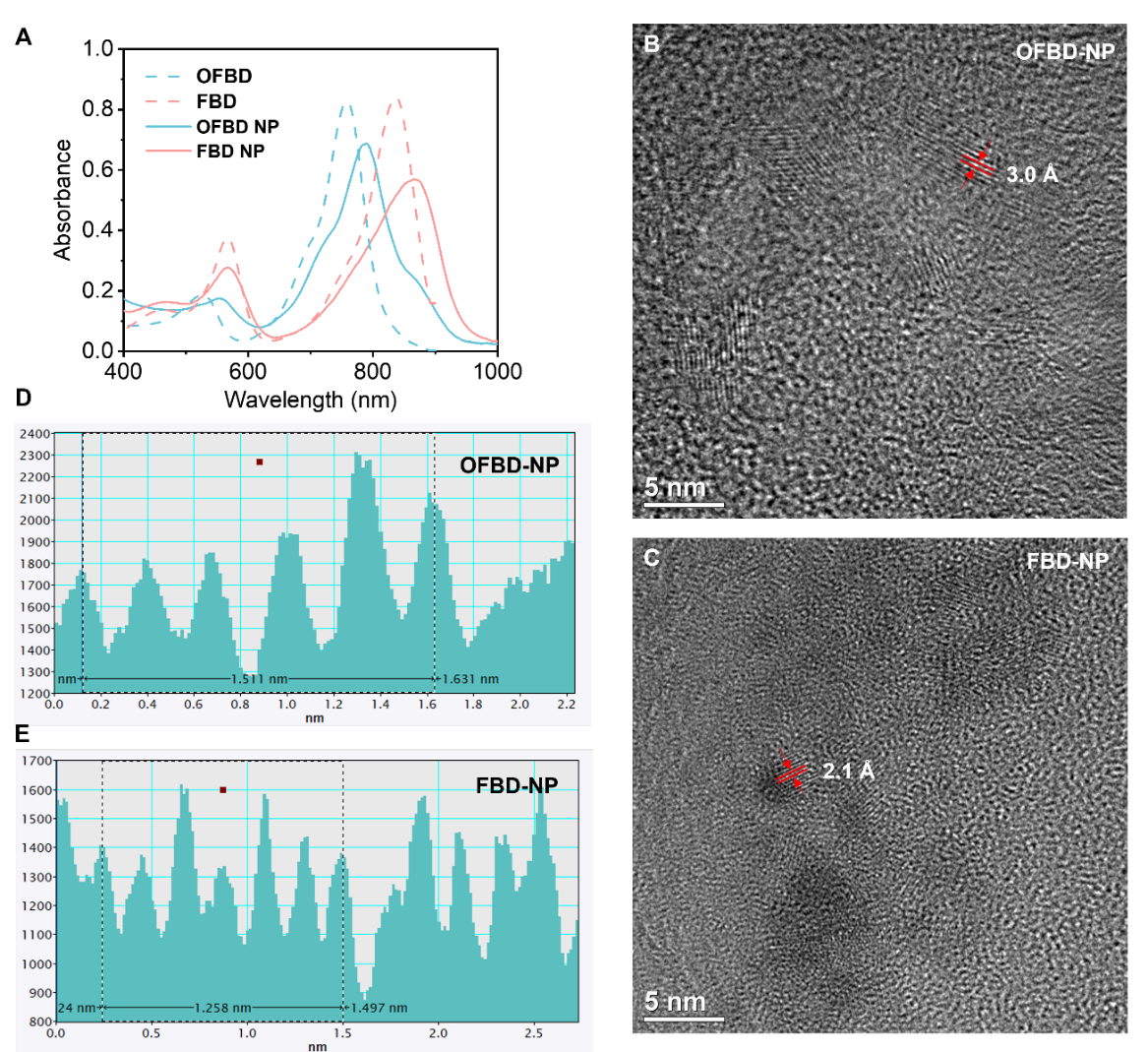


**Figure S8**. Absorption of **OFBD**, **FBD**, and their corresponding nanoemulsions (A). HRTEM images of **OFBD-NP** and **FBD-NP** (B,C). Corresponding lattice fringe spacing analysis (D,E).


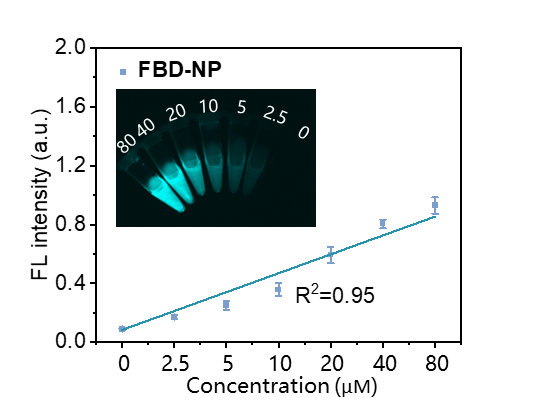


**Figure S9**. FL intensity versus the concentration of **FBD-NP** with inset images of solutions.


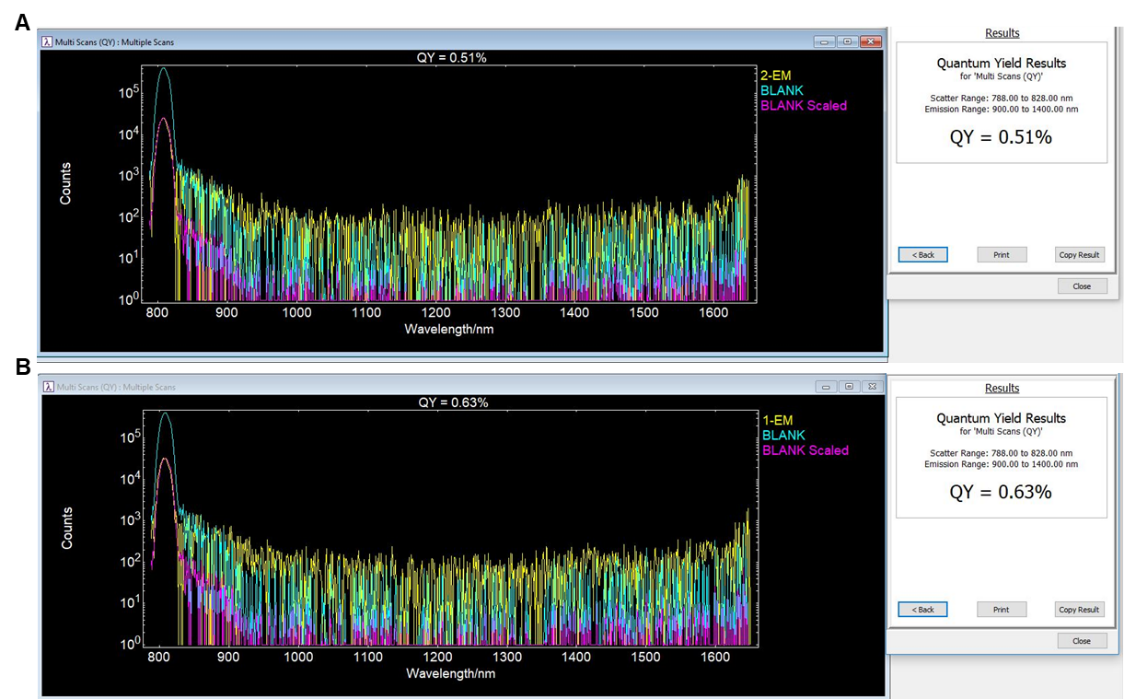


**Figure S10**. Absolute fluorescence quantum yield of **FBD-NP** (A) and **OFBD-NP** (B).


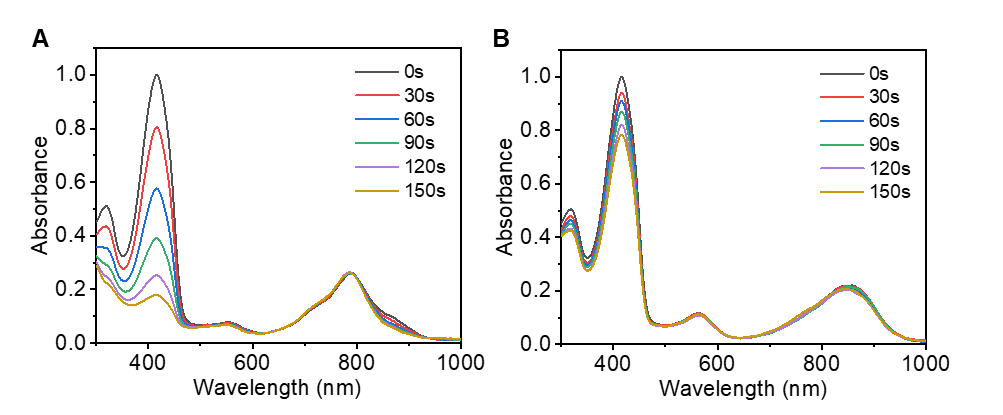


**Figure S11**. Time-dependent absorption spectra of DPBF (60 µM) mixed with either **OFBD-NP** (A, 5 µM) or **FBD-NP** (B, 5 µM) in H_2_O under 808 nm laser irradiation (1 W cm⁻^2^).


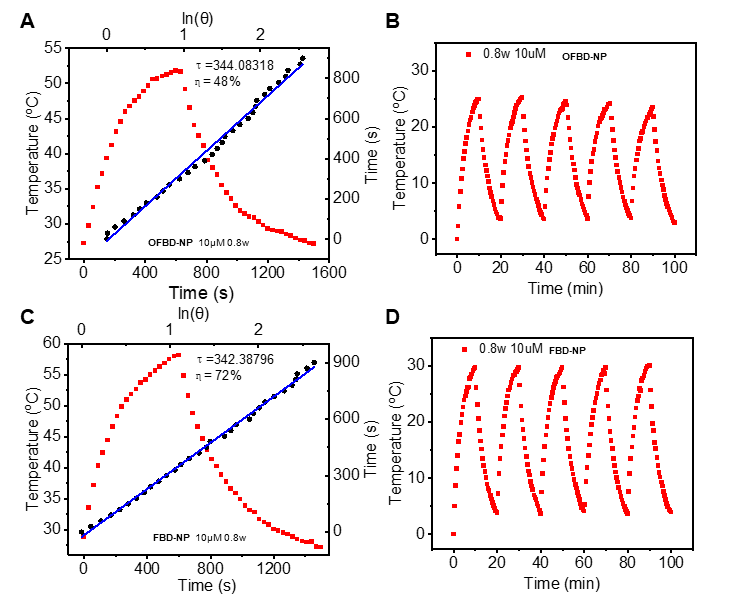


**Figure S12**. Photothermal heating curves and photothermal conversion efficiency (PCE) calculations of **OFBD-NP** (A) and **FBD-NP** (C). Temperature profiles of 10 μM **OFBD-NP** (B) and **FBD-NP** (D) during five irradiation-cooling cycles.


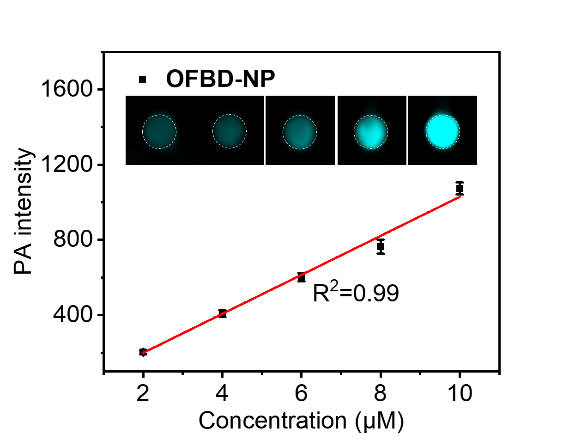


**Figure S13**. Plot of PA signal intensity versus concentration of **OFBD-NP** with inserted PAI images.


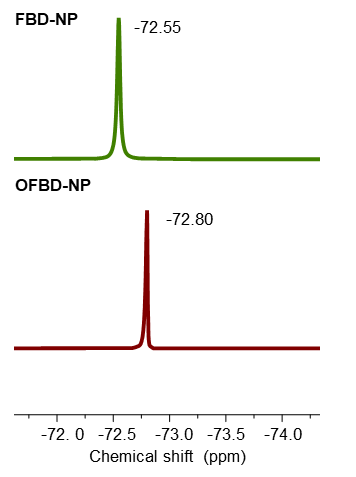


**Figure S14**. Partial ^19^F NMR spectra of **FBD-NP** and **OFBD-NP**.


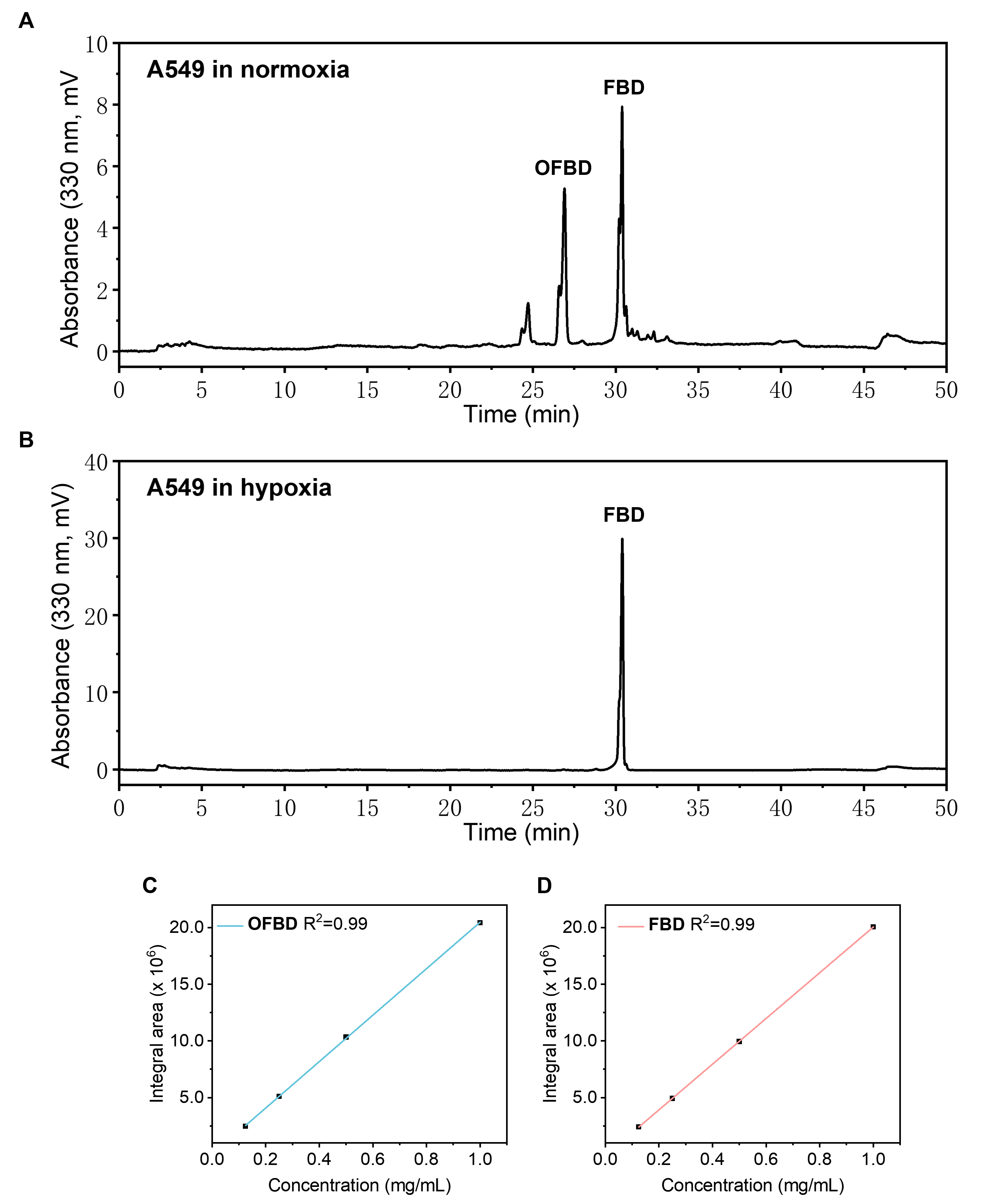


**Figure S15**. HPLC analysis of cell lysates after incubation with **OFBD-NP** under normoxic (21% O_2_) and hypoxic (10% O_2_) conditions (A,B). Standard curves of **OFBD** and **FBD** used for quantification (C,D).


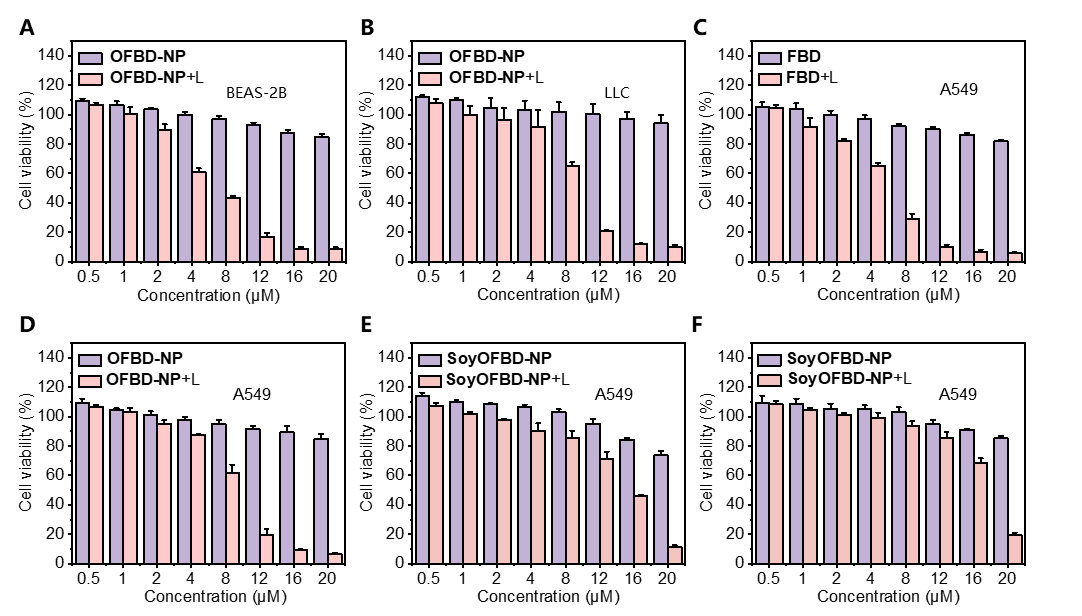


**Figure S16**. Cytotoxicity assay of **OFBD-NP** against BEAS-2B (A), and LLC (B) cells in the dark or after laser irradiation under normoxia. Cytotoxicity assay of **FBD-NP** against A549 cells in the dark or after laser irradiation under normoxia (C). Cytotoxicity assay of **OFBD-NP** against A549 cells in the dark or after laser irradiation under hypoxic (D) conditions. Cytotoxicity assay of **SoyOFBD-NP** against A549 cells in the dark or after laser irradiation under normoxic (E) and hypoxic (F) conditions.


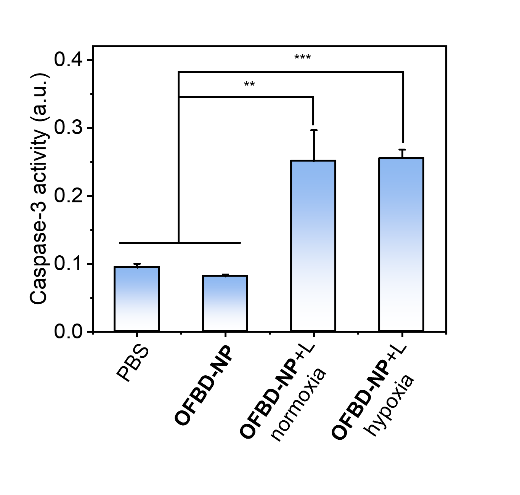


**Figure S17**. Caspase-3 activity assays in A549 cells treated with PBS, **OFBD-NP**, and **OFBD-NP** + laser under normoxic and hypoxic conditions. Data are presented as mean ± SD (n = 3), **p < 0.01, ***p < 0.001.


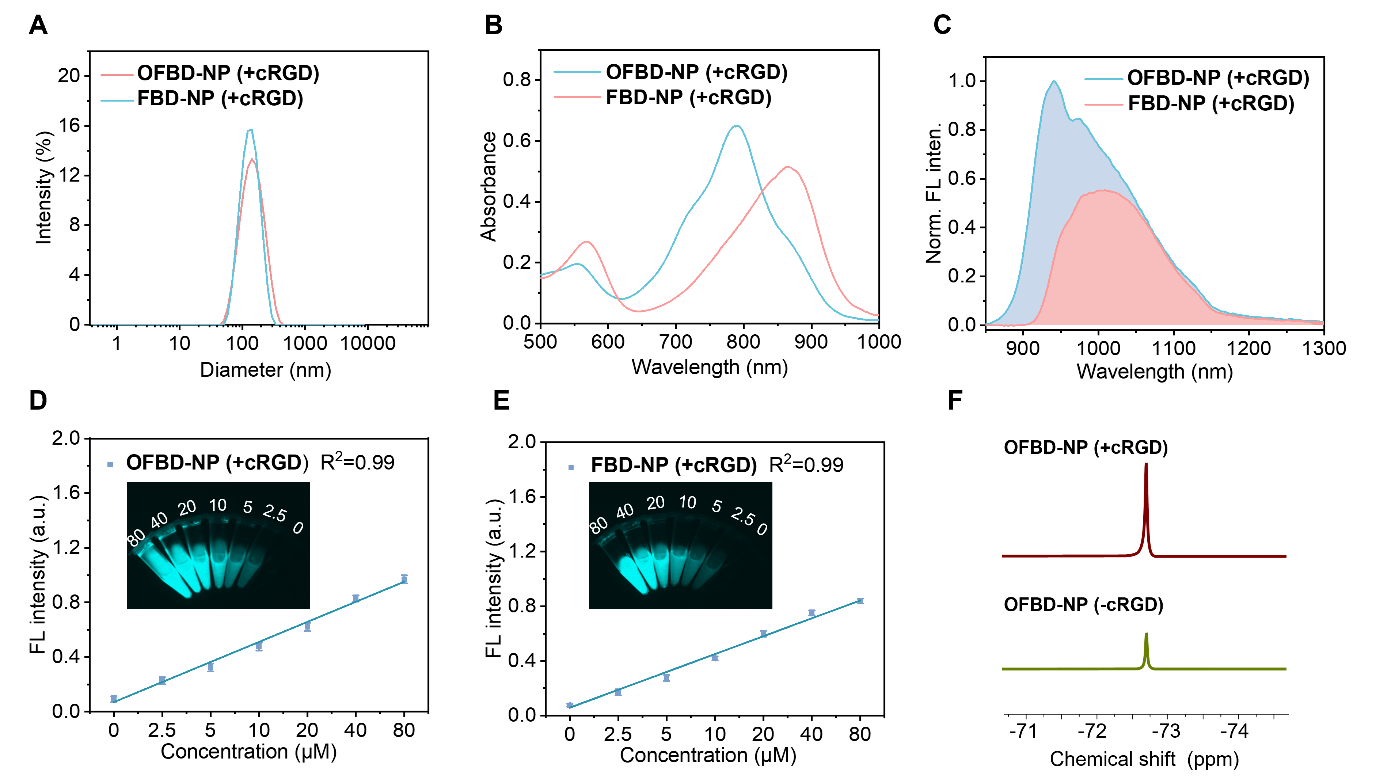


**Figure S18**. DLS (A), absorption (B), normalized fluorescence emission (C), and concentration-dependent fluorescence imaging of cRGD-functionalized **OFBD-NP** and **FBD-NP** (D,E). ^19^F NMR of A549 cell lysates after treatment (F).


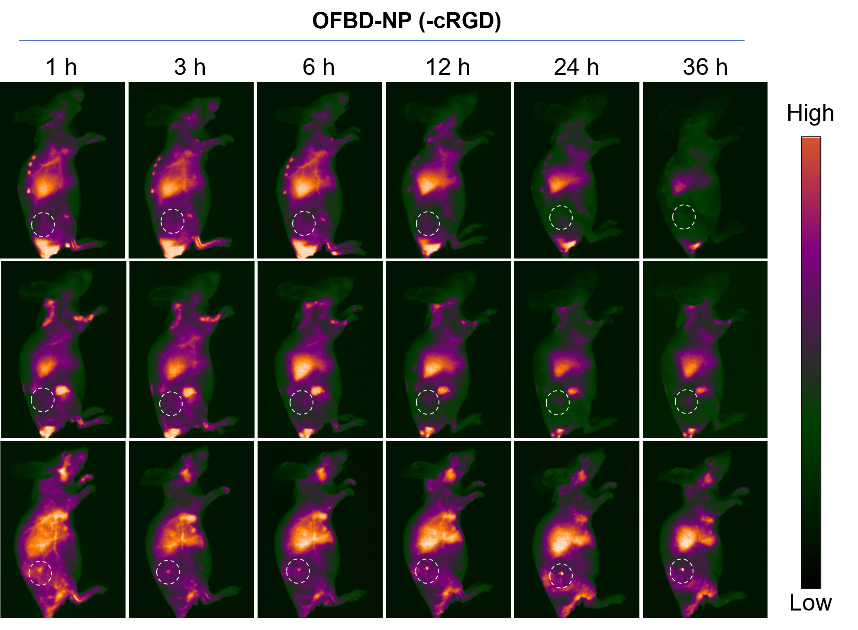


**Figure S19**. Time-dependent NIR-II FLI of mice with subcutaneous tumors after intravenous injection of **OFBD-NP** (without cRGD)


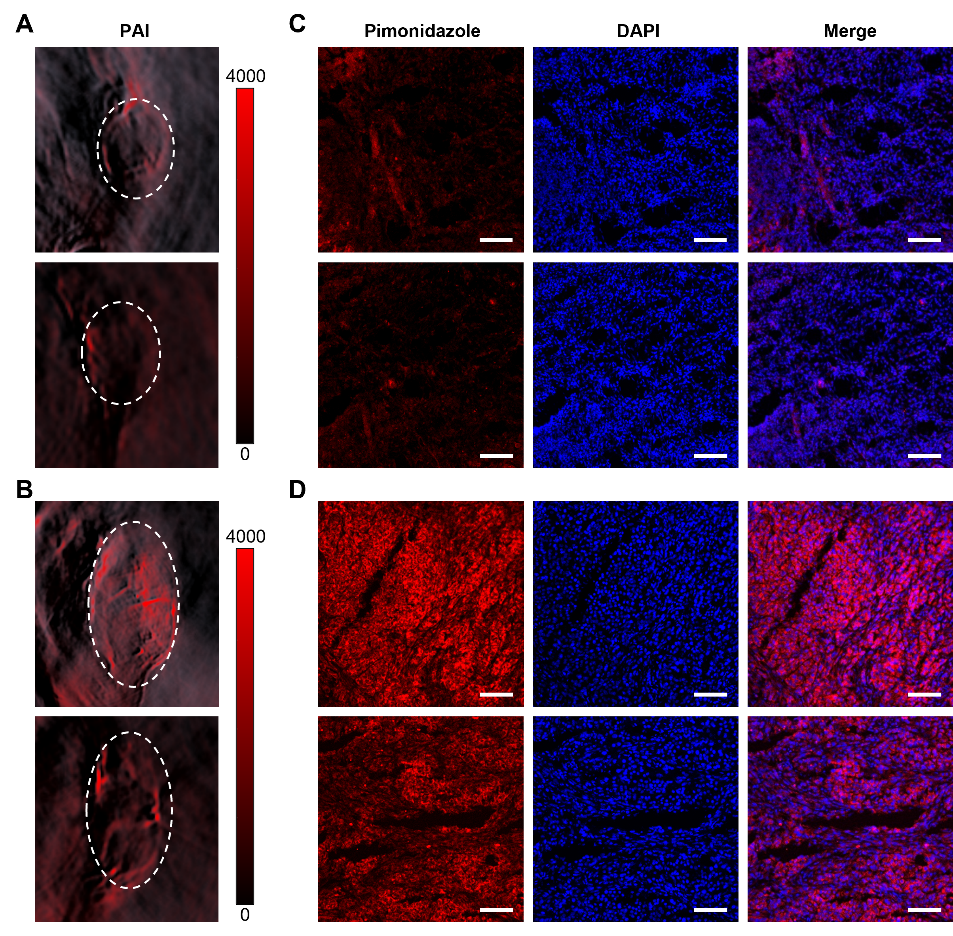


**Figure S20**. Representative PA images of tumors at 12 h with volumes of ~200 mm^3^ and ~400 mm^3^, respectively, shown at two cross-sections (A,B). CLSM images of tumor sections from tumors with volumes of ~200 mm^3^ (C) and ~400 mm^3^ (D) after staining with pimonidazole (Hypoxyprobe Plus Kit) for hypoxia, DAPI for nuclei, and the merged images. Scale bars represent 100 μm.

**Table S1**. Photophysical properties of **OFBD**, **FBD**, **OBD**, **BD**, and their corresponding nanoemulsions.

| 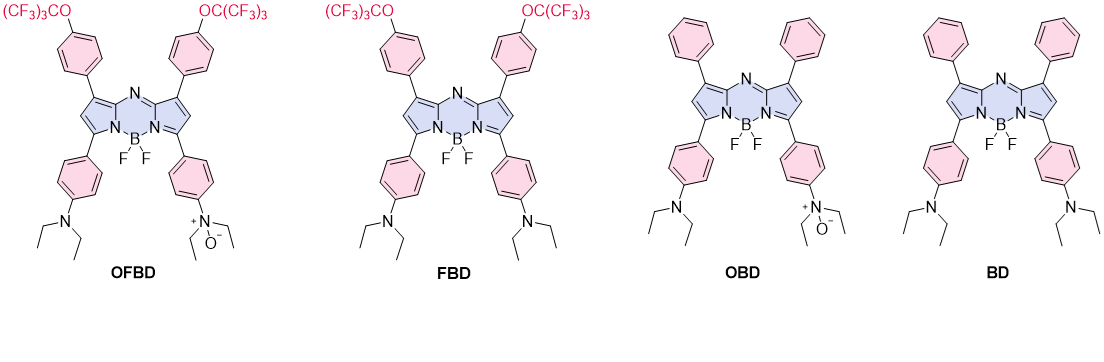 | | | | | |
| --- | --- | --- | --- | --- | --- |
|  | λ_abs_（nm） | λ_em_（nm） | Stoke(nm) | ε  （M^-1^cm^-1^） | ΔT  （^o^C） |
| **OFBD** | 757 | 807 | 50 | 83080 | 16.5 |
| **FBD** | 836 | 971 | 135 | 84050 | 26.5 |
| **OBD*^a^*** | 749 | 800 | 51 | 75550 | / |
| **BD*^a^*** | 817 | 976 | 159 | 65220 | / |
| **OFBD-NP** | 790 | 939 | 149 | / | / |
| **FBD-NP** | 872 | 1007 | 135 | / | / |
| *^a^* Literature data.^[1]^ | | | | | |

**Table S2**. Formulation, size, and PDI of **OFBD-NP**, **FBD-NP**, and **SoyOFBD-NP**.

|  | Lecithin | Cholesterol | DSPE-PEG_2000_ | Photosensitizer | Oil | H_2_O | Size (nm)  PDI |
| --- | --- | --- | --- | --- | --- | --- | --- |
| **OFBD-NP** | 15 mg | 3 mg | 1 mg | **OFBD**  0.6 mg | **Foil**  33 mg | 1 mL | 116.8  0.186 |
| **FBD-NP** | 15 mg | 3 mg | 1 mg | **FBD**  0.6 mg | **Foil**  33 mg | 1 mL | 128.0  0.202 |
| **SoyOFBD-NP** | 15 mg | 3 mg | 1 mg | **OFBD**  0.6 mg | soybean oil  30 mg | 1 mL | 246.0  0.239 |

**Table S3**. Time-dependent cellular uptake of fluorine from **OFBD-NP**.

|  | Incubation time | | | | | |
| --- | --- | --- | --- | --- | --- | --- |
|  | 0 h | 3 h | 6 h | 12 h | 24 h | 36 h |
| Cell number | 2.88×10^6^ | 1.93×10^6^ | 1.98×10^6^ | 2.25×10^6^ | 3.08×10^6^ | 3.15×10^6^ |
|  | 3.33×10^6^ | 2.00×10^6^ | 2.91×10^6^ | 2.68×10^6^ | 3.75×10^6^ | 3.69×10^6^ |
|  | 2.03×10^6^ | 1.96×10^6^ | 2.77×10^6^ | 2.73×10^6^ | 3.26×10^6^ | 2.64×10^6^ |
| ^19^F NMR integral of **OFBD-NP** | 0 | 0.09 | 0.60 | 0.99 | 2.16 | 2.02 |
|  | 0 | 0.15 | 0.49 | 0.90 | 2.38 | 2.54 |
|  | 0 | 0.15 | 0.61 | 0.99 | 1.98 | 1.99 |
| Total internalized fluorine in mol | 0 | 0.45×10^-6^ | 3.00×10^-6^ | 4.95×10^-6^ | 10.80×10^-6^ | 10.10×10^-6^ |
|  | 0 | 0.75×10^-6^ | 2.45×10^-6^ | 4.50×10^-6^ | 11.90×10^-6^ | 12.70×10^-6^ |
|  | 0 | 0.75×10^-6^ | 3.05×10^-6^ | 4.95×10^-6^ | 9.90×10^-6^ | 9.95×10^-6^ |
| Internalized fluorine per cell | 0 | 1.4036×10^11^ | 9.1212×10^11^ | 1.3244×10^12^ | 2.1109×10^12^ | 1.9302×10^12^ |
|  | 0 | 2.2575×10^11^ | 5.0684×10^11^ | 1.0108×10^12^ | 1.9103×10^12^ | 2.0719×10^12^ |
|  | 0 | 2.3036×10^11^ | 6.9895×10^11^ | 1.0915×10^12^ | 1.8281×10^12^ | 2.2689×10^12^ |

^19^F NMR was referenced to CF_3_SO_3_Na (s, -79.6 ppm, 5 mM) in 90% H_2_O and 10% D_2_O.

# 2 Synthesis and Characterization of FBD and OFBD

## 2.1 Synthetic procedures

| 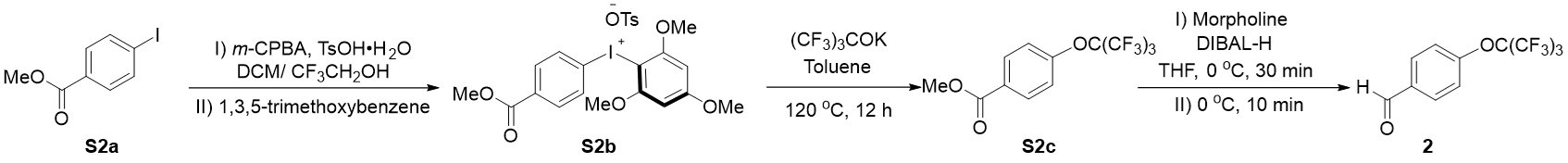  **Scheme S1** Synthetic route of intermediate **2** |
| --- |

**(4-(Methoxycarbonyl)phenyl)(2,4,6-trimethoxyphenyl)iodonium 4-methylbenzenesulfonate** (**S2b**). To a solution of methyl 4-iodobenzoate **S2a** (7.86 g, 30 mmol, 1.0 equiv.) in a mixture of DCM (90 mL) and trifluoroethanol (10 mL) at -20 °C was added *m*-CPBA (7.31 g, 85% w/w, 36 mmol, 1.2 equiv.). The mixture was stirred at -20 °C for 10 min before *p*-toluenesulfonic acid monohydrate (11.43 g, 60 mmol, 2.0 equiv.) was added in one portion. The mixture was slowly heated to 50 °C and stirred until TLC analysis indicated complete consumption of the aryl iodide. The mixture was then cooled to -20 °C, and 1,3,5-trimethoxybenzene (5.54 g, 33 mmol, 1.1 equiv.) was added. Stirring was continued at -20 °C for 5 min. The reaction mixture was removed from the cooling bath and concentrated under reduced pressure. The crude product was dissolved in a minimal amount of MeOH and precipitated in Et_2_O. The precipitate was collected by filtration, washed with Et_2_O, and dried under vacuum to afford **S2b** as a white solid (14.58 g, yield: 81%).^[2]^ ^1^H NMR (500 MHz, DMSO-*d*_6_) δ 8.04 (d, *J* = 8.3 Hz, 2H), 7.96 (d, *J* = 8.3 Hz, 2H), 7.47 (d, *J* = 7.8 Hz, 2H), 7.10 (d, *J* = 7.8 Hz, 2H), 6.48 (s, 2H), 3.94 (s, 6H), 3.87 (s, 3H), 3.85 (s, 3H), 2.28 (s, 3H).

**Methyl 4-((1,1,1,3,3,3-hexafluoro-2-(trifluoromethyl)propan-2-yl)oxy)benzoate** (**S2c**). To a suspension of diaryliodonium salt **S2b** (6.0 g, 10.0 mmol, 1.0 equiv.) in anhydrous toluene (120 mL) was added (CF_3_)_3_COK (7.86 g, 30.0 mmol, 3.0 equiv.). The mixture was stirred at 120 ^o^C until TLC analysis indicated completion. After cooling to room temperature, the mixture was filtered, and the filtrate was concentrated under reduced pressure. The residue was purified by column chromatography (silica gel, PE/EA = 50:1) to afford **S2c** as a colorless liquid (3.0 g, yield: 81%). ^1^H NMR (500 MHz, CDCl_3_) δ 8.05 (d, *J* = 8.9 Hz, 2H), 7.27 (d, *J* = 8.9 Hz, 2H), 3.92 (s, 3H).

**4-((1,1,1,3,3,3-Hexafluoro-2-(trifluoromethyl)propan-2-yl)oxy)benzaldehyde** (**2**). To a round-bottom flask equipped with a stirring bar, morpholine (2.98 mL, 34.0 mmol, 4.2 equiv.) and THF (80 mL) were added under a N_2_ atmosphere at 0 ^o^C. DIBAL-H (32.4 mL, 1.0 M in hexane, 32.4 mmol, 4.0 equiv.) was then added dropwise to the mixture. After stirring for 30 min at 0 ^o^C, compound **S2c** (3.0 g, 8.1 mmol, 1.0 equiv.) was added, and stirring was continued at this temperature until the reaction was complete (as monitored by TLC). The reaction was quenched by the addition of aqueous 1 M HCl solution (80 mL), followed by Et_2_O (150 mL). The mixture was stirred vigorously until the organic layer became transparent. The organic layer was separated, dried over anhydrous MgSO_4_, filtered, and concentrated in vacuo. The resulting crude mixture was purified by silica gel column chromatography using PE: EA = 40:1 as the eluent to afford benzaldehyde **2** as a colorless liquid (1.93 g, yield: 70%).^[3] 1^H NMR (500 MHz, CDCl_3_) δ 10.00 (s, 1H), 7.90 (d, *J* = 8.4 Hz, 2H), 7.36 (d, *J* = 8.4 Hz, 2H). ^13^C NMR (126 MHz, CDCl_3_) δ 189.6, 156.3, 133.9, 130.4, 122.5, 118.9 (q, *J* = 292.3 Hz), 81.3 – 80.1 (m). ^19^F NMR (471 MHz, CDCl_3_) δ -72.17 (s). HRMS (EI): [M-H]^+^ calcd for C_11_H_4_F_9_O_2_^+^: 339.0068, found 339.0062.

The synthesis of all the intermediates is described in the Supporting Information. (*E*)-1-(4-(diethylamino)phenyl)-3-(4-((3,3,3-trifluoroprop-1-yn-1-yl)oxy)phenyl)prop-2-en-1-one--perfluoroethane (**3**). 4'-Dimethylamino acetophenone **1** (1.68 g, 8.78 mmol, 1.1 equiv.) and NaOH (0.64 g, 16.00 mmol, 2.0 equiv.) were dissolved in EtOH. The mixture was stirred for 5 min, and benzaldehyde **2** (2.72 g, 8.00 mmol, 1.0 equiv.) was added in one portion. After stirring for 24 h at room temperature, the reaction mixture was neutralized with 2 M HCl, extracted with EA, dried over anhydrous Na_2_SO_4_ and concentrated in vacuo. The resulting mixture was purified by silica gel column chromatography to afford **3** as a yellow oil (4.00 g, yield: 88%). ^1^H NMR (600 MHz, CDCl_3_) δ 7.98 (d, *J* = 9.0 Hz, 2H), 7.73 (d, *J* = 15.6 Hz, 1H), 7.62 (d, *J* = 8.7 Hz, 2H), 7.55 (d, *J* = 15.6 Hz, 1H), 7.24 (d, *J* = 8.7 Hz, 2H), 6.67 (d, *J* = 9.0 Hz, 2H), 3.44 (q, *J* = 7.1 Hz, 4H), 1.22 (t, *J* = 7.1 Hz, 6H). ^13^C NMR (151 MHz, CDCl_3_) δ 187.1, 153.8, 151.5, 140.5, 134.5, 131.4, 129.4, 125.2, 123.3, 120.1 (q, *J* = 293.5 Hz), 110.3, 82.0 – 81.0 (m), 44.7, 12.7. ^19^F NMR (471 MHz, CDCl_3_) δ -72.23 (s). HRMS (ESI) m/z: [M+Na]^+^ calcd for C_23_H_20_F_9_NNaO_2_^+^: 536.1248, found 536.1240.

1-(4-(diethylamino)phenyl)-3-(4-((1,1,1,3,3,3-hexafluoro-2-(trifluoromethyl)prop-an-2-yl) oxy)phenyl)-4-nitrobutan-1-one (**4**). Compound **3** (3.00 g, 5.84 mmol, 1.0 equiv.), nitromethane (3.57 g, 58.48 mmol, 10.0 equiv.) and KOH (65 mg, 1.16 mmol, 0.2 equiv.) were dissolved in EtOH. The mixture was refluxed for 5 h. After cooling to room temperature, the reaction mixture was neutralized with 2 M HCl, and extracted with DCM. The combined organic layers were dried over anhydrous Na_2_SO_4_, filtered, and concentrated in vacuo. The crude product was purified by flash chromatography to give compound **4** (2.40 g, yield: 71%) as a yellowish oil. ^1^H NMR (500 MHz, CDCl_3_) δ 7.79 (d, *J* = 7.7 Hz, 2H), 7.27 (d, *J* = 7.2 Hz, 2H), 7.15 (d, *J* = 7.2 Hz, 2H), 6.60 (d, *J* = 7.7 Hz, 2H), 4.87 – 4.83 (m, 1H), 4.68 – 4.63 (m, 1H), 4.24 – 4.191 (m, 1H), 3.41 (q, *J* = 7.0 Hz, 4H), 3.30 – 3.27 (m, 2H), 1.19 (t, *J* = 7.0 Hz, 6H). ^13^C NMR (126 MHz, CDCl_3_) δ 194.1, 152.1, 151.6, 138.6, 130.7, 128.9, 123.6, 123.3, 120.0 (q, *J* = 292.95 Hz), 110.3, 80.7 – 81.9 (m), 79.6, 44.7, 40.5, 39.2, 12.5. ^19^F NMR (471 MHz, CDCl_3_) δ -72.25 (s). HRMS (ESI) m/z: [M+Na]^+^ calcd for C_24_H_23_F_9_NNaO_4_^+^: 597.1412, found 597.1409.

4,4'-(5,5-difluoro-1,9-bis(4-((1,1,1,3,3,3-hexafluoro-2-(trifluoromethyl)propan-2-yl) oxy)phenyl)-5H-4l4,5l4-dipyrrolo[1,2-c:2',1'-f][1,3,5,2]triazaborinine-3,7-diyl)bis (N,N-diethyl aniline) (**FBD**). A mixture of **4** (2.90 g, 5.05 mmol, 1.0 equiv.) and ammonium acetate (13.61 g, 176.67 mmol, 35.0 equiv.) was stirred at 120 ^o^C for 5 h. After cooling to room temperature, the reaction mixture was washed with water, extracted with DCM. The combined organic layers were concentrated in vacuo, and the residue was recrystallized from DCM/MeOH to give crude intermediate (0.43 g). This intermediate was used directly in the next step without further purification. Under an argon atmosphere, the above intermediate (0.43 g, 0.40 mmol, 1.0 equiv.) and DIPEA (0.52 g, 4.02 mmol, 10.0 equiv.) were dissolved in dry DCM. The solution was stirred at room temperature for 20 min, followed by the addition of boron trifluoride diethyl etherate BF_3_^.^Et_2_O, (0.81 g, 5.71 mmol, 14.0 equiv.). Stirring was continued at room temperature for 24 h. The reaction was quenched with water and extracted with DCM. The combined organic layers were dried over anhydrous Na_2_SO_4_, filtered, and concentrated in vacuo. The residue was purified by flash chromatography to give **FBD** as a brown solid (423 mg, yield 15%). ^1^H NMR (500 MHz, CDCl_3_) δ 8.13 (d, *J* = 8.8 Hz, 4H), 8.00 (d, *J* = 8.8 Hz, 4H), 7.24 (d, *J* = 8.8 Hz, 4H), 7.08 (s, 2H), 6.73 (d, *J* = 8.8 Hz, 4H), 3.44 (q, *J* = 7.1 Hz, 8H), 1.23 (t, *J* = 7.1 Hz, 12H).^13^C NMR (126 MHz, CDCl_3_) δ 156.0, 153.0, 149.8, 145.2, 138.7, 132.1, 132.0, 130.3, 122.7, 120.2 (q, *J* = 292.8 Hz), 118.3, 118.0, 111.6, 82.5 – 80.6 (m), 44.7, 12.9. ^19^F NMR (471 MHz, CDCl_3_) δ -72.30 (s, 18F), -135.80 – -136.01 (m, 2F). HRMS (ESI) m/z: [M+H]^+^ calcd for C_48_H_39_BF_20_N_5_O_2_^+^: 1107.2908, found 1107.2929.

4-(7-(4-(diethylamino)phenyl)-5,5-difluoro-1,9-bis(4-((1,1,1,3,3,3-hexafluoro-2-(trifluoro methyl)propan-2-yl)oxy)phenyl)-5H-4l4,5l4-dipyrrolo[1,2-c:2',1'-f][1,3,5,2] triazaborinin-3-yl)-*N*,*N*-diethylaniline oxide (**OFBD**). A solution of **FBD** (200 mg, 0.18 mmol, 1.0 equiv.) in DCM was cooled to 0 °C. NaHCO_3_ (17 mg, 0.20 mmol, 1.1 equiv.) and *m*-CPBA (34 mg, 0.20 mmol, 1.1 equiv., 85% purity) were added. The mixture was warmed to room temperature and stirred for 1 h. After quenching with saturated NaHCO_3_, the mixture was extracted with EtOAc. The combined organic layers were dried over anhydrous Na_2_SO_4_ and concentrated in vacuo. The residue was purified by flash chromatography to give **OFBD** as a black solid (144 mg, yield 71%).^47^ ^1^H NMR (500 MHz, CDCl_3_) δ 8.26 (d, *J* = 9.0 Hz, 2H), 8.11 (d, *J* = 8.5 Hz, 2H), 8.02 (d, *J* = 8.9 Hz, 2H), 7.97 (d, *J* = 8.9 Hz, 2H), 7.89 (d, *J* = 8.5 Hz, 2H), 7.34 (s, 1H), 7.28 (d, *J* = 8.5 Hz, 2H), 7.23 (d, *J* = 8.5 Hz, 2H), 6.89 (s, 1H), 6.78 (d, *J* = 9.0 Hz, 2H), 3.80 – 3.64 (m, 4H), 3.51 (q, *J* = 7.14 Hz, 4H), 1.28 – 1.21 (m, 12H). ^13^C NMR (126 MHz, CDCl_3_) δ 161.8, 153.8, 152.6, 152.0, 149.5, 148.3, 142.9, 142.6, 135.1, 134.4, 133.5, 132.4, 130.7, 130.6, 129.9, 129.7, 122.9, 122.8, 122.0, 121.5, 120.1 (q, *J* = 295.5 Hz), 116.3, 116.0, 112.2, 84.0 – 79.8 (m), 67.0, 45.1, 12.9, 8.6. ^19^F NMR (471 MHz, CDCl_3_) δ -72.30 (s, 9F), -72.33 (s, 9F), -135.71 – -135.92 (m, 2F). HRMS (ESI) m/z: [M+H]^+^ calcd for C_48_H_39_BF_20_N_5_O_3_^+^: 1124.2827, found 1124.2826.

**1,11-bis((1,1,1,3,3,3-hexafluoro-2-(trifluoromethyl)propan-2-yl)oxy)undecane** (**Foil)**. **Foil** was synthesized in our previous work.^[4]^ ^1^H NMR (500 MHz, CDCl_3_) δ 3.99 (t, *J* = 6.5 Hz, 4H), 1.71-1.62 (m, 4H), 1.40-1.36 (m, 4H), 1.32-1.29 (m, 10 H).

## 2.2 Photothermal performance and photostability of OFBD and FBD

The temperature changes of the **OFBD** and **FBD** in CHCl_3_ (10 µM) under 808 nm laser irradiation (0.5 W cm^-2^) for 10 min were monitored using a thermal camera. The temperature data represent the average value of three replicate experiments.

The photostability of **OFBD** and **FBD** (4 μM) was assessed by comparing their absorption spectra recorded before and after irradiation (808 nm, 0.5 W cm^-2^). For comparison, the photostability of ICG (4 μM) was evaluated under identical conditions as the control.

## 2.3 Detection of singlet oxygen generation via DPBF degradation

The singlet oxygen (^1^O_2_) generation capabilities of **OFBD** and **FBD** were evaluated using DPBF as a chemical probe. Separate solutions containing each compound (2 μM) and DPBF (60 μM) in CHCl_3_ were irradiated with an 808 nm laser (0.5 W cm⁻^2^). The time-dependent degradation of DPBF was monitored via the decrease in absorbance ratios at 415 nm to quantify ^1^O_2_ production. Results represent the mean of three independent experiments.

## 2.4 Biotransformation of OFBD under normoxic and hypoxic conditions

Phosphate buffer (pH 7.4) was degassed by bubbling with nitrogen for 30 min before use. **OFBD** was dissolved in deionized water containing 0.1% (v/v) cremophor EL (CrEL) and incubated with liver microsomes (LM, 200 μg/mL) and NADPH (100 μg/ml) at 37 °C. Incubations were carried out under either normoxic (21% O_2_) or hypoxic (N_2_ atmosphere) conditions and terminated at predetermined time points (0, 0.5, 1, 2, 4, 6, 12, and 18 h). After incubation, the reaction mixture was extracted with DCM, and the organic phase was collected and concentrated under reduced pressure. The residue was redissolved in chloroform, and UV-vis absorption spectra were recorded to evaluate hypoxia-dependent spectral changes associated with the biotransformation of **OFBD**.

## 2.5 Preparation of nanoemulsions

Preparation of **OFBD-NP**:

Egg yolk lecithin (15 mg), cholesterol (3 mg), DSPE-PEG_2000_ (1 mg), **Foil** (30 mg), and **OFBD** (0.6 mg) were dissolved in DCM and evaporated under vacuum. Subsequently, 1 mL of deionized water was added, followed by sonication for 15 min. The mixture was then extruded 15 times through a 200 nm carbonate membrane filter to obtain **OFBD-NP**.

For the preparation of cRGD-functionalized nanoemulsions, DSPE-PEG_2000_ was replaced with DSPE-PEG_2000_-cRGD (2 mg/mL), and **OFBD-NP** (+cRGD) was obtained following the same procedure. **FBD-NP** were prepared analogously using **FBD** in place of **OFBD.**

Preparation of **SoyOFBD-NP**:

Egg yolk lecithin (15 mg), cholesterol (3 mg), DSPE-PEG_2000_ (1 mg), soybean oil (25 mg), and **OFBD** (0.6 mg) were dissolved in DCM. To this solution, 1 mL of deionized water was added. After stirring for 4 h, the mixture was subjected to sonication for 15 min and allowed to stand for DCM evaporation. **SoyOFBD-NP** were obtained by filtering the resulting dispersion through a 0.22 µm carbonate membrane filter.

## 2.6 Determination of photothermal conversion efficiency

A nanoemulsion sample with a concentration of 10 μM photosensitizer was irradiated under an 808 nm (0.8 W cm^-2^) laser for 10 min, then the sample temperature was measured every 30 s until it naturally cooled down to ambient temperature. The photothermal conversion efficiency (η) of the sample can be calculated according to the following equation:

$\eta=\frac{hs\left( T_{max}-T_{surr} \right)-Q_{dis}}{I(1-{10}^{-A_{\lambda}})}$ (1)

Where h is the heat transfer coefficient, s is the surface area of the container, T_max_ is the equilibrium temperature, T_surr_ is the ambient temperature (25.1 ℃), I is the laser power used for the photothermal experiment (0.8 W cm^-2^), A_λ_ is the absorbance at the used laser wavelength (808 nm), and η is the photothermal transduction efficiency. Q_dis_ represents the heat dissipated from the laser through the solvent and the container.

The value of Q_dis_ can be obtained using the following equation:

$Q_{dis}=hs\left( T_{max,H2O}-T_{surr} \right)$ (2)

Where T_max, H2O_ is the maximum temperature of water.

The value of *hs* can be obtained using the following equation:

$hs=\frac{m_{i}C_{i}}{\tau_{s}}$ (3)

where m_i_ is the mass of water, C_i_ is the heat capacity of water (4.2 J/g), τ_s_ is the sample system time constant. To determine τ_s_, a dimensionless driving force temperature θ is introduced, which is defined by the following equation:

$t=\tau_{s}(-ln\theta)$ (4)

$\theta=\frac{T_{surr}-T}{T_{surr}-T_{max}}$ (5)

Where T is the real-time temperature of the sample when the laser was turned off, t represents the time.

## 2.7 *In vitro* ^19^F MRI

The ^19^F MRI phantom study was performed using a 9.4T scanner (Bruker) with a RARE (rapid acquisition with refocused echoes) sequence. Phantom samples were prepared with varying concentrations of **OFBD** or **FBD** in CHCl_3_ (40 mM, 20 mM, 10 mM, and 5 mM). ^19^F MR images were acquired using the following parameters: center frequency = 376.526758 MHz, repetition time (TR) = 1000 ms, echo time (TE) = 3 ms, field of view (FOV) = 30 mm × 30 mm, slice thickness (SI) = 20 mm, matrix size = 32 × 32, RARE factor = 4, number of averages (NS) = 32. The total acquisition time of 256 s.

# 3 Cell culture

The cells were cultured in a cell incubator at 37 °C and 5% CO_2_ using Dulbecco’s modified eagle medium (DMEM) (high glucose) medium supplemented with 10% (v/v) fetal bovine serum and 1% (v/v) penicillin-streptomycin.

## 3.1 Intracellular uptake

Confocal Laser Scanning Microscopy (CLSM). A549 cells were seeded into 35 mm confocal dishes at a density of 1.5 × 10^5^ cells per well and incubated overnight. The cells were incubated with OFBD-NP for varying durations (0, 3 h, 6 h, 12 h, 24 h, and 36 h). Following incubation, the cells were washed three times with PBS and fixed with 4% paraformaldehyde (PFA) for 15 min. After staining with 200 µL of DAPI for 10 min, images were captured using CLSM.

^19^F Nuclear Magnetic Resonance (NMR) Analysis. A549 cells were seeded in 6-well plates at a density of 1 × 10^6^ cells per well and cultured for 24 h to allow complete attachment. Cells were then incubated with OFBD-NP for varying durations (0, 3 h, 6 h, 12 h, 24 h, and 36 h). After incubation, the cells were harvested, collected in EP tubes, washed three times with PBS, and lysed using RIPA buffer for 30 min. The ^19^F NMR spectra of the lysates were subsequently recorded on a Bruker 500 MHz spectrometer.

## 3.2 Cellular bioreduction in A549 cells

A549 cells were seeded in 10 cm culture dishes and allowed to adhere under normoxic (21% O_2_) or hypoxic (10% O_2_) conditions. The cells were then incubated with OFBD-NP (+cRGD) for 12 h under the corresponding oxygen conditions. After incubation, cells were collected, washed three times with PBS, and lysed using RIPA buffer for 15 min. The lysates were treated with tetrahydrofuran to facilitate the dissolution of OFBD and its reduced products, followed by HPLC analysis to quantify the conversion of OFBD to FBD. In parallel, cell pellets collected after incubation were used directly for photoacoustic imaging to assess hypoxia-dependent signal changes.

## 3.3 *In vitro* cytotoxicity assays

The cytotoxicity of **OFBD-NP** against A549, LLC, and BEAS-2B cell lines was evaluated using the CCK-8 assay. Cells were seeded into 96-well plates at a density of 1 × 10⁴ cells per well and incubated overnight at 37 °C under normoxic or hypoxic conditions to allow adherence. Subsequently, the cells were incubated with varying concentrations of **OFBD-NP** for 24 h. Following incubation, the cells were irradiated with an 808 nm laser (1 W cm^-2^, 6 min) and cultured for an additional 24 h. Afterward, 100 µL of fresh medium containing 10% (v/v) CCK-8 was added to each well, followed by incubation at 37 °C for 1 h. Absorbance at 450 nm was measured using a microplate reader to assess the cell viability. For dark toxicity measurement of the photosensitizers, the same procedure was followed without laser irradiation. All data are presented as mean ± SD, n ≥ 3.

## 3.4 Live/dead cell staining assays

A549 cells were seeded onto 35 mm confocal dishes at a density of 1 × 10^5^ cells per dish and incubated with **OFBD-NP** (C**_OFBD_** = 20 µM) for 24 h under normoxic or hypoxic conditions. Following exposure to an 808 nm laser (1 W cm^-2^) for 6 min, the cells were incubated for an additional 6 h. The cells were then stained with Calcein-AM and propidium iodide (PI) for 30 min. Confocal fluorescence imaging was subsequently conducted. To assess the dark toxicity, an identical procedure was performed without laser irradiation.

## 3.5 Intracellular ROS imaging in A549 cells

Intracellular reactive oxygen species (ROS) generation in A549 cells was detected using 2',7'-dichlorodihydrofluorescein diacetate (DCFH-DA). The cells were seeded onto 35 mm confocal dishes and incubated with **OFBD-NP** (C**_OFBD_** = 20 µM) for 24 h. Subsequently, cells were stained with 10 μM DCFH-DA for 30 min. After washing three times with PBS, the laser-treated group was irradiated with an 808 nm laser (1 W cm^-2^) for 6 min. Confocal fluorescence imaging was then performed (λ_ex_ = 488 nm).

## 3.6 Hemolysis assay

The red blood cells (RBCs) were isolated from BALB/c mice. Whole blood was centrifuged at 1500 rpm for 5 min, and washed 4 times with PBS (pH 7.4) until the supernatant became clear. RBC suspension in 0.5 mL PBS was then mixed with 0.5 mL **OFBD-NP** solutions at varying concentrations (0.4, 0.8, 1.6, 3.1, 6.2, 12.5, 25, 50, and 100.0 μM). PBS and deionized water served as negative and positive controls, respectively. Then, the obtained suspension was cultured at 37 °C for 3 h and centrifuged to observe the color of the supernatant. Finally, the absorption of the supernatant was measured at 541 nm using an ultraviolet spectrophotometer. The hemolysis percentage values were calculated via the formula:

Hemolysis (%) = (A_s_ – A_n_)/(A_p_ – A_n_),

where the A_s_ represented the sample absorbance, A_p_ represented the positive control absorbance, and A_n_ represented the negative control absorbance. The experiment for each sample was repeated three times.

## 3.7 Apoptosis assay

A549 cells were seeded in 6-well plates at a density of 1 × 10^6^ cells per well and cultured for 24 h to achieve full attachment. The cells were treated with OFBD-NP (C_OFBD_ = 20 µM) for 24 h under normoxic or hypoxic conditions. Following treatment, the cells were irradiated with 808 nm laser (1 W cm^-2^) for 6 min. After 6 h incubation, cells were harvested, collected in EP tubes, and washed twice with cold PBS (centrifugation: 1500 rpm, 5 min, 4°C). Cell pellets were resuspended in 100 μL of binding buffer. Cells were stained with 5 μL of Annexin V-FITC and 10 μL PI for 15 min at 4 °C in the dark. Before flow cytometry analysis, samples were filtered with FalconTM cell strainers. Unstained cells served as a negative control.

# 4 *In vivo* Assays

**Animals and tumor model**. All animal experiments were conducted in accordance with the Guidelines for Animal Care and Use of the Innovation Academy for Precision Measurement Science and Technology, Chinese Academy of Sciences. Female BALB/c nude mice (5 weeks old) were purchased from Hubei BIONT Biological Technology Co., Ltd. and maintained on an SPF-grade diet. To establish the A549 xenograft tumor model, A549 cells (1 × 10^7^) suspended in 0.1 mL of PBS were injected subcutaneously into the flank of female BALB/c nude mice. Tumor volume (V) was calculated using the formula:

V = (*L* × *W*× *W)/2*

Where *L* represents the longest tumor diameter (length) and *W* represents the perpendicular shorter diameter (width).

## 4.1 Bioreduction of OFBD in vivo

Tumor-bearing mice with tumor volumes of approximately 200 mm^3^ and 400 mm^3^ were selected and intravenously injected with **OFBD-NP** (+cRGD) via the tail vein. Photoacoustic imaging (PAI) was performed 24 h post-injection to monitor hypoxia-dependent bioreduction of **OFBD** in tumors of different sizes.

## 4.2 Assessment of tumor hypoxia in tumor tissues

Tumor hypoxia levels were evaluated using the Hypoxyprobe Plus Kit. Following photoacoustic imaging, mice were intravenously injected with pimonidazole hydrochloride (100 μL, 12 mg mL^-1^). The mice were then sacrificed, and tumor tissues were harvested, embedded, and cryosectioned into 20 μm slices. Tumor sections were stained with a FITC-labeled mouse IgG1 monoclonal antibody (FITC-Mab1) according to the manufacturer’s protocol and imaged by confocal laser scanning microscopy (CLSM). Fluorescence signals from pimonidazole adducts were collected in the 500-550 nm range under 488 nm excitation.

## 4.3 *In vivo* NIR-II fluorescence imaging

Mice bearing A549 tumors were intravenously injected with 100 µL of **OFBD-NP** (C**_OFBD_** = 2 mg/kg). Biodistribution of **OFBD-NP** was monitored using a NIR-II fluorescence imaging system (Series II 900/1700, NIROPTICS, China) at 1, 3, 6, 12, 24, and 36 h post-injection.

## 4.4 *In vivo* ^19^F MRI

Mice bearing A549 tumors received intravenous injections of 200 µL of OFBD-NP (C_OFBD_ = 4 mg/kg, C_F_ = 9 mmol/kg). ^19^F MRI was performed using the RARE sequence with the following parameters: center frequency = 376.526778 MHz, TR = 1500 ms, TE = 3 ms, FOV = 37 mm × 37 mm, SI = 15 mm, matrix size = 32 × 32, rare factor = 8, and NS = 600.

Anatomical reference ^1^H MR images were acquired using the RARE sequence with the following parameters: TR = 2500 ms, TE = 11 ms, FOV = 30 mm × 30 mm, SI = 1 mm, matrix size = 256 × 256, rare factor = 8, and NS = 4.

## 4.5 *In vivo* PAI

Mice bearing A549 tumors were intravenously injected with 100 µL of **OFBD-NP** (C**_OFBD_** = 2 mg/kg). Tumor PA intensity was measured at 1, 3, 6, 12, 24, and 36 h post-injection.

## 4.6 Therapeutic efficacy evaluation

When the subcutaneous tumors reached approximately 100 mm^3^, the mice were randomly divided into six groups: (a) saline; (b) saline + L; (c) OFBD-NP (2 mg/kg); (d) OFBD-NP (2 mg/kg) + L; (e) FBD-NP (2 mg/kg); (f) FBD-NP (2 mg/kg) + L. The mice received intravenous injections of OFBD-NP on days 0 and 3, followed by irradiation with an 808 nm laser (0.8 W cm^-2^, 6 min) 12 h post-injection. The body weight and tumor volume of mice were recorded every 2 days. After 20 days of treatment, the mice were euthanized, and the major organs and tumors were harvested for H&E staining.

# 5 Statistical analysis

Data are presented as mean ± standard deviation from n ≥ 3 replicates. Statistical significance was evaluated using an unpaired two-sided Student’s *t*-test, with p values calculated in Microsoft Excel. Asterisks indicate significant differences (*p < 0.05, **p < 0.01, ***p < 0.001).

# 6 ^1^H/^19^F/^13^C NMR and HRMS spectra of compounds

Compound **S2b**, ^1^H NMR (500 MHz, DMSO-*d_6_*)

Compound **S2c**, ^1^H NMR (500 MHz, CDCl_3_)

Compound **S2c**, ^13^C NMR (126 MHz, CDCl_3_)

Compound **S2c**, ^19^F NMR (471 MHz, CDCl_3_)

Compound **2**, ^1^H NMR (500 MHz, CDCl_3_)

Compound **2**, ^13^C NMR (126 MHz, CDCl_3_)

Compound **2**, ^19^F NMR (471 MHz, CDCl_3_)

HRMS spectra of compound **2**


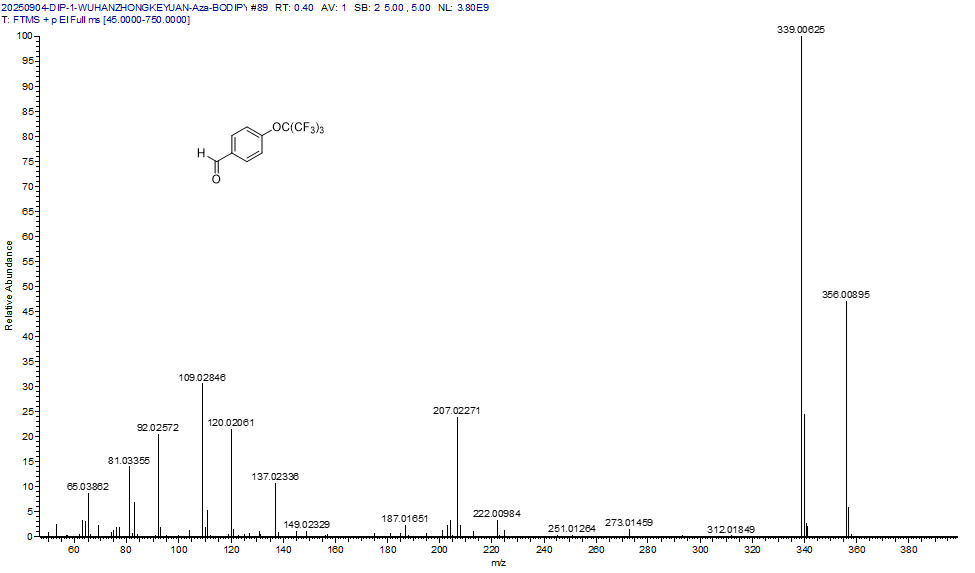


Compound **3**, ^1^H NMR (600 MHz, CDCl_3_)

Compound **3**, ^13^C NMR (151 MHz, CDCl_3_)

Compound **3**, ^19^F NMR (471 MHz, CDCl_3_)

HRMS spectra of compound **3**


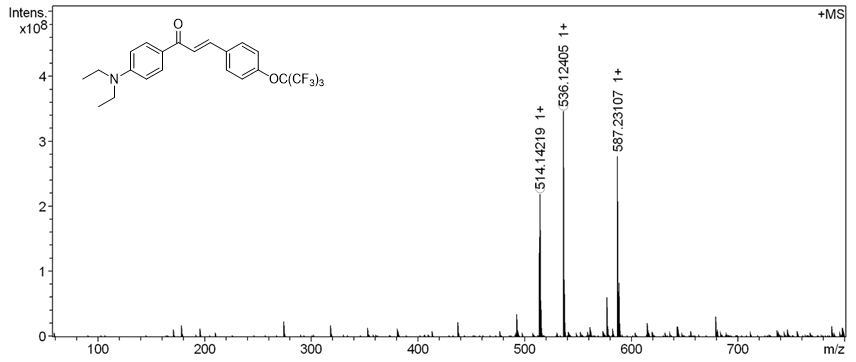


Compound **4**, ^1^H NMR (500 MHz, CDCl_3_)

Compound **4**, ^13^C NMR (126 MHz, CDCl_3_)

Compound **4**, ^19^F NMR (471 MHz, CDCl_3_)

HRMS spectra of compound **4**

**
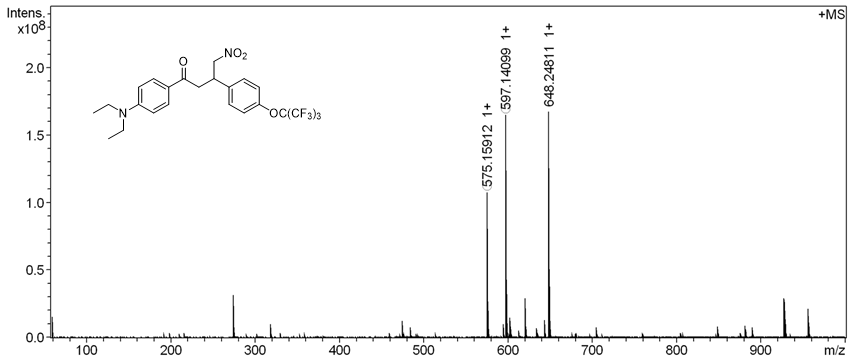
**

**FBD**, ^1^H NMR (500 MHz, CDCl_3_)

**FBD**, ^13^C NMR (126 MHz, CDCl_3_)

**FBD**, ^19^F NMR (471 MHz, CDCl_3_)

HRMS spectra of **FBD**


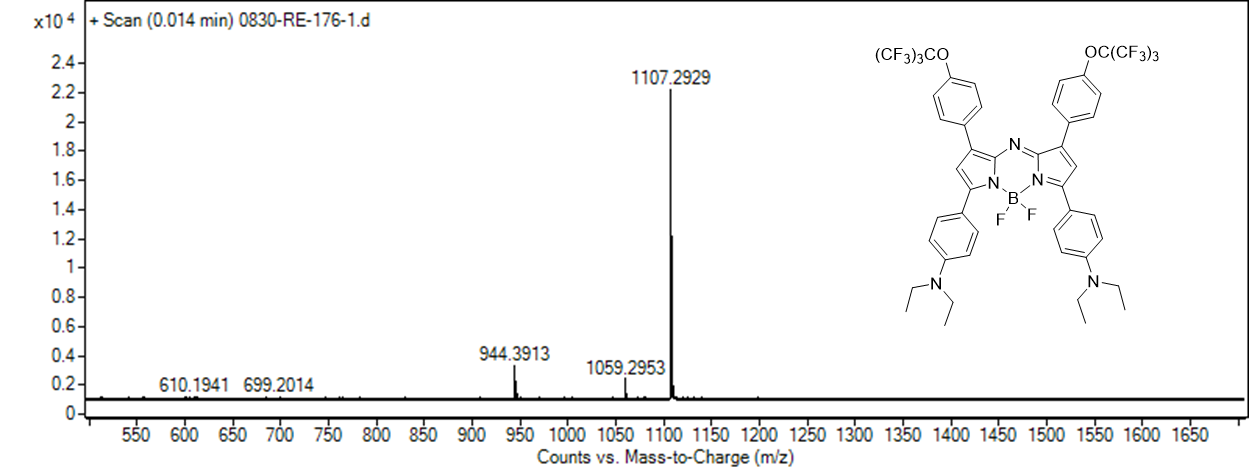


HPLC spectrum of **FBD**

**
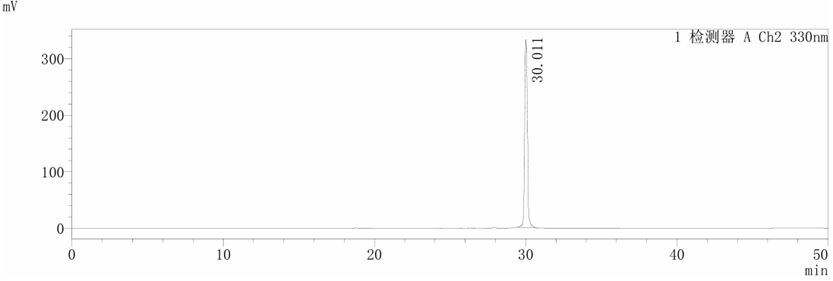
**

| No. | Retention Time | Area | Peak height | Percent |
| --- | --- | --- | --- | --- |
| 1 | 30.011 | 4215520 | 332281 | 100 |

**OFBD**, ^1^H NMR (500 MHz, CDCl_3_)

**OFBD**, ^13^C NMR (126 MHz, CDCl_3_)

**OFBD**, ^19^F NMR (471 MHz, CDCl_3_)


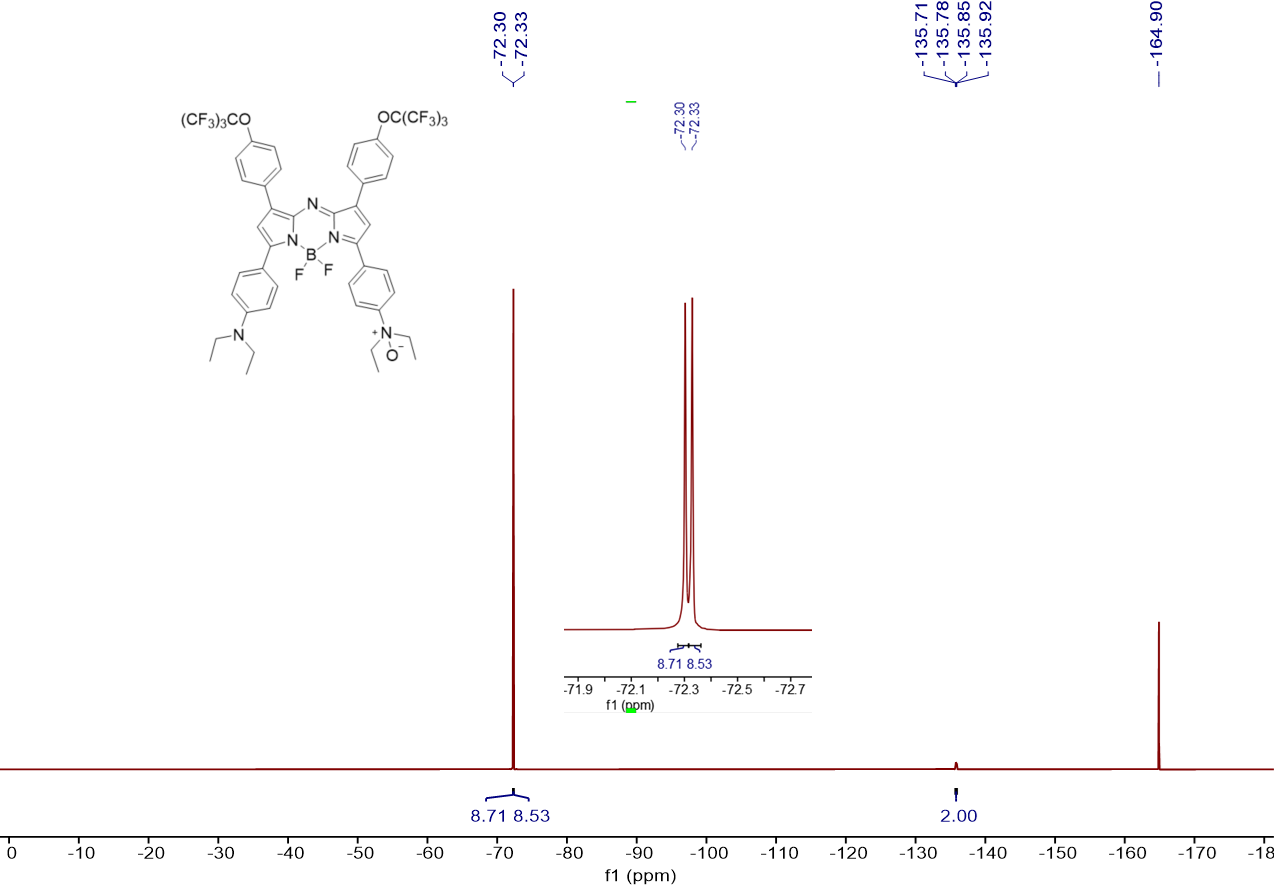


HRMS spectra of **OFBD**


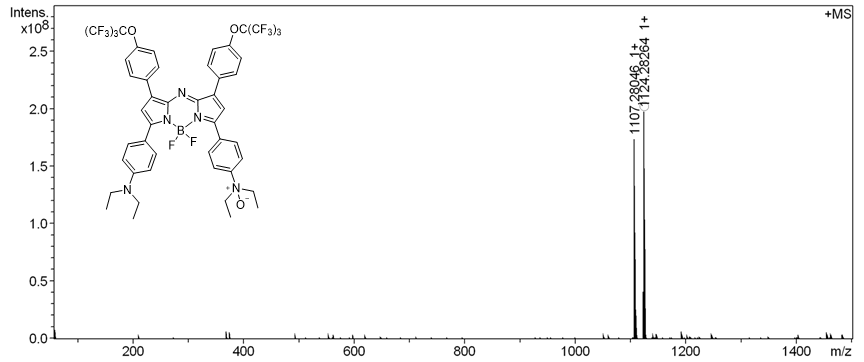


HPLC spectrum of **OFBD**


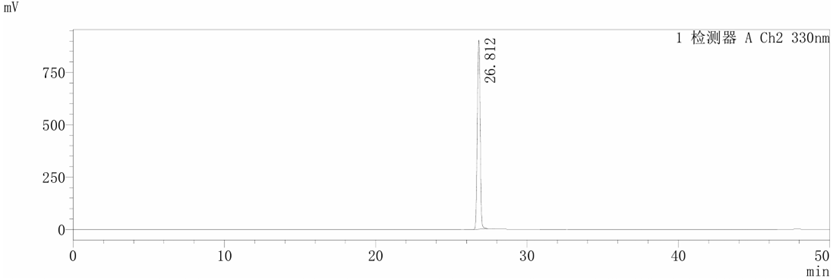


| No. | Retention Time | Area | Peak height | Percent |
| --- | --- | --- | --- | --- |
| 1 | 26.812 | 11780780 | 902246 | 100 |

**Foil**, ^1^H NMR (500 MHz, CDCl_3_)


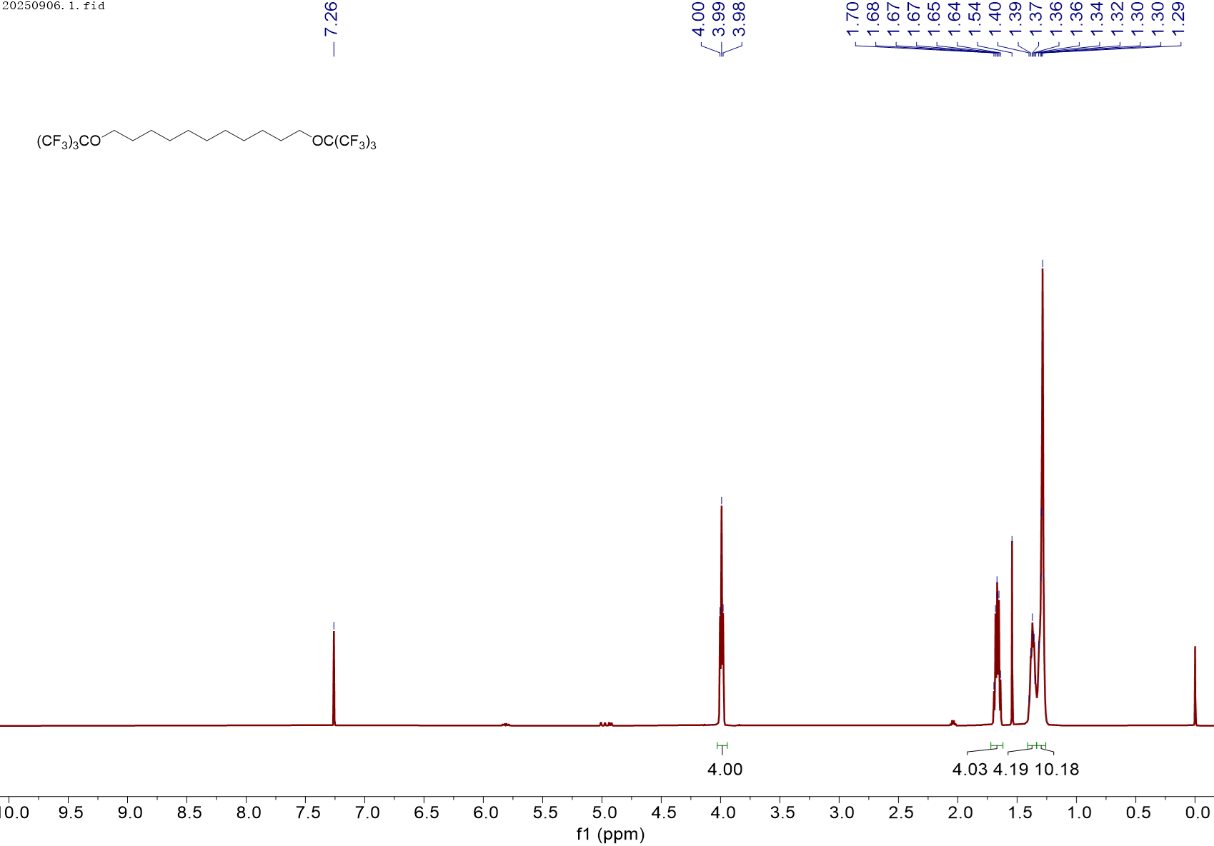


**7 References**

[1] H. J. Knox, T. W. Kim, Z. Zhu, J. Chan, *ACS Chem. Biol.* **2018**, *13*, 1838-1843.

[2] H. Meng, L. Wen, Z. Xu, Y. Li, J. Hao, Y. Zhao, *Org. Lett.* **2019**, *21*, 5206-5210.

[3] S. Young Kim, Y. Ri Kim, H. Tae Kim, A. Kumar Jaladi, D. Keun An, *ChemistrySelect* **2022**, *7*, e202202351.

[4] Y. Li, J. Zhang, L. Zhu, M. Jiang, C. Ke, H. Long, R. Lin, C. Ye, X. Zhou, Z. X. Jiang, S. Chen, *Adv. Healthcare Mater.* **2023**, *12*, e2300941.
